# Immunocytochemical Localization of Enzymes Involved in Dopamine, Serotonin, and Acetylcholine Synthesis in the Optic Neuropils and Neuroendocrine System of Eyestalks of *Paralithodes camtschaticus*

**DOI:** 10.3389/fnana.2022.844654

**Published:** 2022-04-08

**Authors:** Elena Kotsyuba, Vyacheslav Dyachuk

**Affiliations:** A.V. Zhirmunsky National Scientific Center of Marine Biology, Far Eastern Branch, Russian Academy of Sciences, Vladivostok, Russia

**Keywords:** serotonin, acetylcholine, tyrosine hydroxylase, sinus gland, dopamine, king crab, crustacea

## Abstract

Identifying the neurotransmitters secreted by specific neurons in crustacean eyestalks is crucial to understanding their physiological roles. Here, we combined immunocytochemistry with confocal microscopy and identified the neurotransmitters dopamine (DA), serotonin (5-HT), and acetylcholine (ACh) in the optic neuropils and X-organ sinus gland (XO-SG) complex of the eyestalks of *Paralithodes camtschaticus* (red king crab). The distribution of Ach neurons was studied by choline acetyltransferase (ChAT) immunohistochemistry and compared with that of DA neurons examined in the same or adjacent sections by tyrosine hydroxylase (TH) immunohistochemistry. We detected 5-HT, TH, and ChAT in columnar, amacrine, and tangential neurons in the optic neuropils and established the presence of immunoreactive fibers and neurons in the terminal medulla in the XO region of the lateral protocerebrum. Additionally, we detected ChAT and 5-HT in the endogenous cells of the SG of *P. camtschaticus* for the first time. Furthermore, localization of 5-HT- and ChAT-positive cells in the SG indicated that these neurotransmitters locally modulate the secretion of neurohormones that are synthesized in the XO. These findings establish the presence of several neurotransmitters in the XO-SG complex of *P. camtschaticus*.

## Introduction

Similar to other arthropods, crustaceans possess a highly developed visual system and exhibit conspicuous visually guided behaviors (Zeil and Hemmi, 2006; Tomsic et al., 2017) in foraging (Tomsic et al., 2017), prey and mate recognition (Murai and Backwell, 2006), defensive strategies (Hemmi, 2005; Hemmi and Tomsic, 2012), spatial orientation, and environmental evaluation (Medan et al., 2015). The red king crab *Paralithodes camtschaticus* (Tilesius, 1815) is a commercially valuable species belonging to the Anomura group of the order Decapoda and inhabits the Bering Sea, Sea of Japan, and Sea of Okhotsk, as well as the North Pacific from the Kamchatka Peninsula to Alaska (Donaldson and Byersdorfer, 2005; Stevens and Lovrich, 2014). An invasive population of this species occurs in the Barents Sea (Dvoretsky and Dvoretsky, 2015, 2018). Additionally, *P. camtschaticus* is a shelf species found in habitats at depths of up to ∼300 m (Pavlova et al., 2007; Dvoretsky and Dvoretsky, 2013, 2015).

The optic lobes of *P. camtschaticus* and other decapod crustaceans are located within the eyestalks and connect with the brain *via* the protocerebral tract. In the eyestalks of decapods and insects, primary visual processing is conducted in retinotopic neuropils referred to as the lamina, medulla, lobula, and lobula plate (Sinakevitch et al., 2003; Sztarker et al., 2005; Harzsch and Hansson, 2008; Krieger et al., 2012; Ito et al., 2014). As observed in insects, optic neuropils in crustaceans are connected *via* two chiasmata: one connects the lamina to the medulla, and the other connects the medulla to the lobula. The optic lobe also comprises other regions that form the lateral protocerebrum (Harzsch and Hansson, 2008; Maza et al., 2021; Strausfeld, 2021). To date, the neural organization and cellular morphologies of the crustacean optic neuropils have been studied in few taxa, including entomostracans and malacostracans, in the mysid *Neomysis integer* (Strausfeld and Nässel, 1981), euphausiacean *Meganyctiphanes norvegica* (Strausfeld and Nässel, 1981), isopod *Ligia occidentalis* (Sinakevitch et al., 2003), stomatopods (Strausfeld and Nässel, 1981; Thoen et al., 2017), and various decapods (Elofsson and Dahl, 1970; Nässel, 1977; Stowe et al., 1977; Sinakevitch et al., 2003; Sztarker et al., 2005, 2009; Sztarker and Tomsic, 2014). Although the neural organization and cellular morphologies of optic neuropils have been studied in these crustaceans, those in the optic neuropils of the red kind crab remain to be elucidated.

The key neuroendocrine center located in the eyestalks is the X-organ sinus gland (XO-SG) complex (Andrew and Saleuddin, 1978; Fingerman, 1992, 1997; Christie, 2011), which secretes hormones that regulate blood sugar levels and the molting, growth, and breeding processes of crustaceans (Cooke and Sullivan, 1982; Webster and Keller, 1987; Allayie et al., 2011; Pérez-Polanco et al., 2011; Chen et al., 2020). Furthermore, it is a crucial regulator of pigment migration in both the retina and chromatophores (De Kleijn and Van Herp, 1995) and also regulates the ability of crustaceans to metabolically adapt to changing environmental conditions (Chung et al., 2010). Despite progress in elucidating crustacean endocrinology in the previous decade, the neurochemical organization of the crustacean eyestalk remains poorly understood. Topographical data on the neurotransmitters that regulate neurohormone synthesis and release is important for the development of aquaculture technology. This is particularly critical for the adjustment of hormonal regulation in commercially valuable species, such as king crabs (Dvoretsky et al., 2021).

The expression and release of neurohormones in the XO-SG complex is regulated by neurons or factors secreted by peripheral cells and tissues and that relay signals that encode information about the internal and external environments (Aréchiga et al., 1985; Christie, 2011). Moreover, studies report that environmental and endogenous factors, such as light, dark, stressful stimuli, and circadian rhythms, affect this expression and release (Aréchiga et al., 1985; García and Aréchiga, 1998). For example, the expression and release of neuropeptides, such as the red pigment-concentrating hormone and the crustacean hyperglycemic hormone (CHH), in the XO-SG complex follows a circadian rhythm, as the complex is driven by retinal illumination (Glantz et al., 1983). Furthermore, hormones secreted by the XO-SG complex regulate the circadian rhythm depending on the adaptation of the eyes and body to light and the environment (Aréchiga et al., 1985; Rao, 2001; Aréchiga and Rodriguez-Sosa, 2002). These effects are reportedly mediated by various neurotransmitters and modulators (Fingeman and Nagabushanam, 1992); however, the mechanisms underlying the interaction between neurons of the visual system and the XO-SG complex in crustaceans are poorly understood.

The morphology of and relationships between the neural elements of the optic lobes of crabs have been comprehensively studied, and the neural elements reportedly demonstrate a highly ordered retinotopic organization (Sztarker et al., 2005, 2009). studies on several crustacean species (Harzsch and Hansson, 2008; Wolff et al., 2012; Krieger et al., 2015; Maza et al., 2016, 2021; Sayre and Strausfeld, 2019; Strausfeld et al., 2020; Strausfeld, 2021) report detailed neuroanatomical descriptions of highly ordered centers in the eyestalks. In previous decades, a variety of neuroactive substances, including serotonin (5-HT), dopamine (DA), GABA, acetylcholine (ACh), and various neuropeptides, such as enkephalin, substance P, molt-inhibiting hormone, red pigment-concentrating hormone, and CHH, have been identified in the crustacean brain and eyestalks by electrophoresis, high-performance liquid chromatography, and immunocytochemistry (Hildebrand et al., 1974; Mancillas et al., 1981; Cooke and Sullivan, 1982; Elofsson et al., 1982; Beltz and Kravitz, 1983; Elofsson, 1983; Nassel et al., 1985; Schueler et al., 1986; Siwicki and Bishon, 1986; Mangerich and Keller, 1988; Rudolph and Spaziani, 1990; Beltz, 1999; Sullivan and Beltz, 2004; Polanska et al., 2007, 2012; Santhoshi et al., 2008; Christie, 2011; Pérez-Polanco et al., 2011; Stewart et al., 2013; Rajendiran and Vasudevan, 2016).

Additionally, 5-HT, DA, GABA, FMRFamide, and substance P have been detected and attributed to single neurons in the optic neuropils and lateral protocerebrum of Stomatopoda (Thoen et al., 2017, 2019) and a few anomuran and brachyuran species (Krieger et al., 2010, 2012; Wolff et al., 2012; Strausfeld et al., 2020; Strausfeld, 2021). Furthermore, multiple studies have demonstrated that neurotransmitters can modulate visual information processing in arthropods (Crow and Bridge, 1985; Kloppenburg and Erber, 1995; Chen et al., 1999; Cheng and Frye, 2020), and various neurotransmitters reportedly regulate the release of neuropeptides from the XO-SG complex (Fingerman, 1997; Saenz et al., 1997; Lee et al., 2000; Alvarez Alvarado et al., 2005; Pitts and Mykles, 2015).

Despite numerous studies, there is little information about neurotransmitters, especially the Ach lobe of the optic nerve. The most accurate marker of cholinergic neurotransmission is antibodies against choline acetyltransferase (ChAT), an enzyme involved in Ach biosynthesis (Yasuyama and Salvaterra, 1999) and encoded by the *Cha* gene (Itoh et al., 1986).

Moreover, the physiological roles and neuroarchitectures of cells synthesizing neurotransmitters in the optic and neuroendocrine centers of crustacean eyestalks are only partially known. Neurons can be mediated by more than one neurotransmitter, and a neurotransmitter can exert varying and even opposing effects on neurons (Nusbaum et al., 2001; Marder and Thirumalai, 2002; Birren and Marder, 2013). Thus, mapping neurotransmitters and co-transmitters and studying their functional interactions and roles in the optic and neuroendocrine centers is a necessity. Therefore, in this study, we analyzed the distribution of neurons expressing markers for 5-HT, DA, and ACh in the eyestalks of *P. camtschaticus*.

## Materials and Methods

### Preparation of Animal Tissue Samples

We captured adult male red king crabs (Tilesius, 1815) measuring ∼150 mm in carapace width in the northwestern Pacific Ocean. Thereafter, the animals were kept in aerated seawater tanks at a temperature of 5 ± 0.5°C, a salinity of 30–31%, and a water-soluble oxygen concentration of 8.1–8.5 mg/L under natural light–dark cycles. During the 2 weeks of adaptation, the water in the tanks was changed three times weekly, and the animals were fed fresh blue mussels (*Mytilus edulis*) once every 2 days. Subsequently, the animals were anesthetized for at least 1 h on ice, and their eyestalks and supraesophageal ganglion were immediately dissected and fixed. These procedures were conducted in accordance with the European Community Council Directive of November 24, 1986 (86/609/EEC). All possible efforts were taken to minimize the number of animals used in this study.

### Immunohistochemical Analysis

The eyestalk and supraesophageal ganglion were fixed with 4% paraformaldehyde dissolved in phosphate-buffered saline (PBS; pH 7.4) for 2 h at 4°C. The fixed samples were washed several times with PBS and incubated overnight in 30% sucrose (prepared in PBS) at 4°C for cryoprotection. Thereafter, the specimens were embedded in the optimum cutting temperature medium Cryomount (Cat. 45830; HistoLab, Espoo, Finland), frozen, and cut into 25–35-μm serial sections using a Cryo-Star HM560 MV cryostat (Thermo Fisher Scientific, Waltham, MA, United States). These sections were mounted on slides and coated with poly-L-lysine (Sigma, St. Louis, MO, United States), after which they were air-dried and stored at −20°C for subsequent staining. We performed immunohistochemical staining of ChAT, tyrosine hydroxylase (TH), and 5-HT in the serial transverse sections through the eyestalks. We used a coordinate system previously proposed for crabs, wherein the eyestalk was oriented at 90° to the horizontal plane (Sztarker et al., 2005).

For immunohistochemical staining, the freshly frozen sections were processed, as described previously (Dyachuk et al., 2015). To eliminate nonspecific binding, the slides were incubated overnight in a blocking buffer comprising 10% normal donkey serum, 1% Triton-X 100, and 1% bovine serum albumin (BSA; Millipore, Burlington, MA, United States) dissolved in 1 × PBS at 4°C. Additionally, we dissolved the following polyclonal primary antibodies in this buffer: rabbit anti-TH (1:500; Millipore, Burlington, MA, United States), rabbit or goat anti-5-HT (1:2000; ImmunoStar Inc., Hudson, WI, United States), and goat anti-ChAT (1:500; Millipore, Burlington, MA, United States). Moreover, a primary mouse anti-synapsin antibody (1:500; clone 3C11; Developmental Studies Hybridoma Bank, Iowa City, IA, United States) was also used, as previously described (Krieger et al., 2012). Subsequently, the sections were washed in 0.01 M PBS (pH 7.4) containing 0.5% Triton X-100 (pH 7.4) prior to incubation with 488-, 555-, or 647-Alexa Fluor-conjugated donkey secondary antibodies (1:1000; Invitrogen, Thermo Fisher Scientific, Waltham, MA, United States) along with the nuclear marker 4′,6-diamidino-2-phenylindole (DAPI; Sigma-Aldrich) for 2 h at 22°C. The sections were then washed with PBS and embedded in glycerol (Merck, Kenilworth, NJ, United States).

### Primary Antibody Specificity and Immunohistochemical Control

We used polyclonal rabbit or goat antibodies that targeted BSA-bound 5-HT with paraformaldehyde (Cat. Nos. 20080 and 20079, respectively; ImmunoStar Inc., Hudson, United States). According to manufacturer instructions, staining with these antibodies is completely eliminated upon pretreatment with 25 μg of the 5-HT-BSA conjugate per 1 mL of diluted antibody. We demonstrated that overnight preincubation of the antibody with 10 μg/mL of the conjugate (Cat. No. 20081; ImmunoStar Inc., Hudson, United States) at 4°C completely eliminated 5-HT immunolabeling in our control tissues. Furthermore, overnight preadsorption of the diluted antibody with 10 mg/mL BSA at 4°C did not affect this staining (i.e., these antibodies recognized 5-HT alone and not BSA). This 5-HT antibody has been used to detect 5-HT in arthropod brains, including that of crabs, hermit crabs, and lobsters (Beltz and Kravitz, 1983; Elofsson, 1983; Harzsch and Waloszek, 2000; Sayre and Strausfeld, 2019).

The rabbit anti-TH antibody (Cat. No. AB152; Millipore, Burlington, MA, United States) targets TH as a key enzyme involved in tyrosine biosynthesis. The antibody against TH was previously identified in the eyestalk ganglia of the blue crab *Callinectes sapidus* (Wood and Derby, 1996) and also in that of the crab *Neohelice granulata* (Klappenbach et al., 2012; Maza et al., 2021). Previous immunohistological studies on related crustaceans demonstrated that antibodies against DA and TH yield highly consistent staining patterns (Cournil et al., 1994; Wood and Derby, 1996), thereby validating the use of a TH antibody as a reliable marker of dopaminergic neurons in crustaceans. Cytoplasmic ChAT, which synthesizes acetylcholinesterase (AChE), is a more specific marker of cholinergic neurons than AChE itself. In this study, we utilized the manufacturer-recommended concentration anti-ChAT along with a concentrated blocking buffer to eliminate nonspecific binding. Antibodies against these proteins are used as phenotypic markers for cholinergic neurons (Salvaterra and Kitamoto, 2001). We performed anti-ChAT labeling similar to that for TH and 5-HT, with the exception of the primary antibody. Polyclonal goat anti-ChAT (Cat. No. AB143; Millipore, Burlington, MA, United States) was diluted to 1:500 in blocking buffer, and we followed a recently published protocol for ChAT immunostaining (Kotsyuba and Dyachuk, 2021). To avoid nonspecific immunorecognition, we performed immunohistochemistry by bypassing the use of primary antibodies and used only secondary antibodies (1:500–1000; I5006, I5381, and I5256; Sigma, St. Louis, MO, United States). We cut and stained at least 30 tissue sections of the eyestalks for each combination of the immunolabels. To visualize the neural structures of the eyestalks, these sections were incubated with mouse monoclonal anti- synapsin, which targets a presynaptic marker (SYNORF1 or antibody 3C11). A previous study showed that this antibody detects an epitope widely conserved in the nervous systems of arthropods, including that of crustaceans (Harzsch et al., 1997; Beltz et al., 2003; Harzsch and Hansson, 2008; Krieger et al., 2012, 2015). The sections were then incubated with 10 mg/mL of the nuclear marker DAPI (in PBS) following pre-incubation with the secondary antibody.

### Microscopy and Imaging

Images for immunohistochemistry were captured with a Zeiss LSM 700 confocal microscope (Carl Zeiss, Oberkochen, Germany) and analyzed using ImageJ software (National Institutes of Health, Bethesda, MD, United States). This software was used for three-dimensional visualization and analysis of the confocal stacks. Each section was sequentially scanned for each fluorophore, and separate and overlaid (of all three channels) images were obtained, which were subsequently converted into projected images using subsets of z-stacks. The converted images were saved as TIFF-images and transferred to Photoshop CS software (Adobe, San Jose, CA, United States), and their contrast and brightness were adjusted for optimal clarity. Negative controls for each fluorochrome were scanned using the same settings.

### Nomenclature

The neuroanatomical nomenclature used in this study is based on that proposed by Sandeman et al. (1992, 1993), with some modifications adopted from Harzsch and Hansson (2008) and Richter et al. (2010). Additionally, we classified the major cell types and neuropils in the *P. camtschaticus* eyestalks according to previously described classifications in the eyestalks of several decapod species (Wang-Bennett and Glantz, 1987a,b; Sztarker et al., 2005; Krieger et al., 2010, 2012; Sztarker and Tomsic, 2014). In this study, we did not study the distribution of tested neurotransmitters in the hemiellipsoid bodies.

## Results

### General Neuromorphology of *Paralithodes camtschaticus* Eyestalks

**FIGURE 1:**
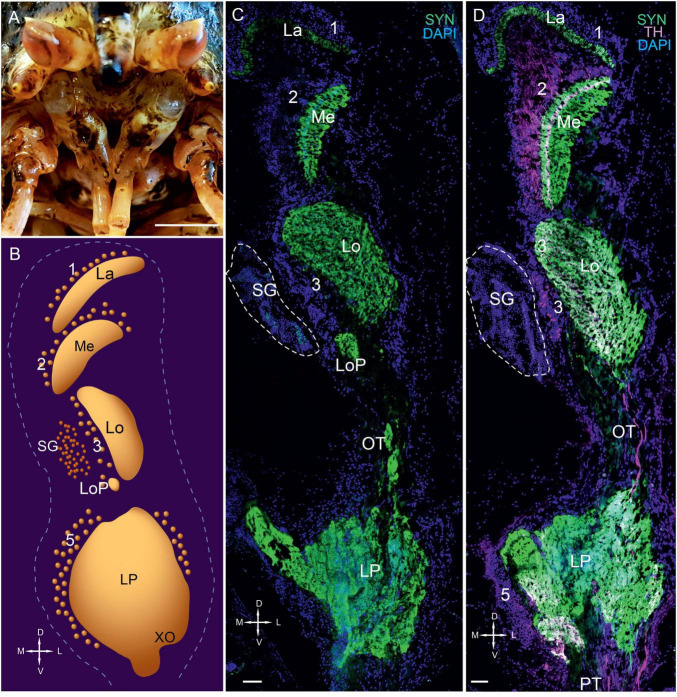
Eyestalks of the red king crab *Paralithodes camtschaticus*. **(A)** Photographs demonstrating the position of the eyestalks in *P. camtschaticus*. **(B)** Diagram of the optic neuropils, SG, and LP (dorsal view). **(C)** SYN detected in the optic neuropils and LP of the dorsal to ventral sections. **(D)** SYN and TH detected in optic neuropils, LP, and OT. Green, SYN; magenta, TH. Dashed lines in panels **(C,D)** indicate cells corresponding to the SG. Scale bars = **(A)** 1 cm and **(C)** 100 μm. La, lamina; Me, medulla; Lo, lobula; LoP, lobula plate; 1, 2, 3, 5, cell clusters; OT, optic tract; LP, lateral protocerebrum; SYN, synapsin; D, dorsal; V, ventral; L, lateral; M, medial; A, anterior; P, posterior.

We determined that the compound eyes of *P. camtschaticus* were vertically elongated ellipsoids located on the eyestalks at the front of the carapace (Figure 1A). The eyestalks had an average height and width of 12.3 ± 1.1 mm and 6.4 ± 0.5 mm, respectively. Similar to other decapods (Harzsch and Hansson, 2008; Krieger et al., 2010, 2012), the eyestalks, optic neuropils (lamina, medulla, lobula, and lobula plate), SG, and lateral protocerebrum of *P. camtschaticus* were arranged from the periphery to the center beneath the retina, respectively (Figures 1B,C). All neuropils were identified by detecting immunolabeled synapsin (Figures 1C,D). In the lamina, immunolabeled synapsin was detected in a thin layer that corresponded to the plexiform layer (Figure 1D). The monopolar neuronal somata (cell cluster 1) were localized above the lamina.

**FIGURE 2:**
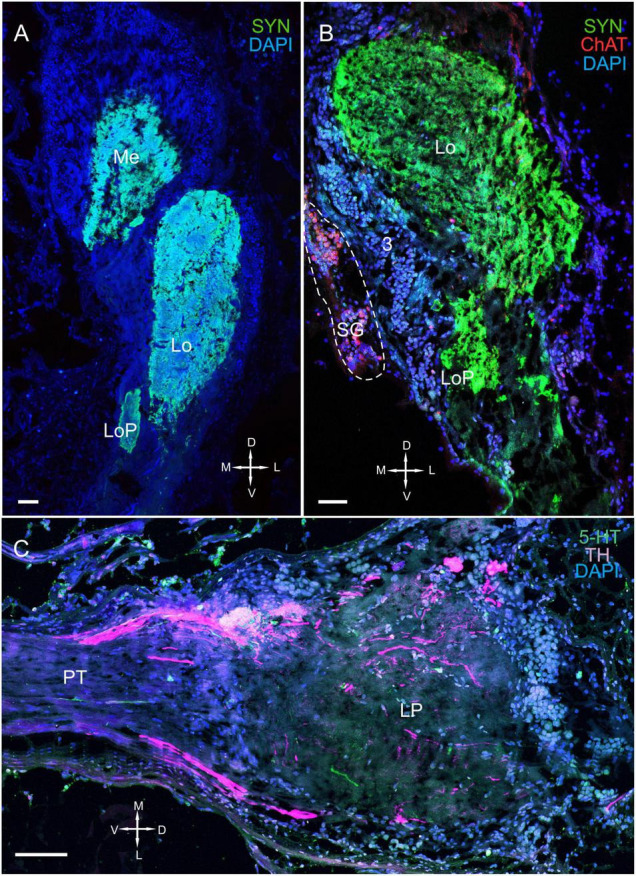
Immunolabeled optic neuropils, SG, and lateral protocerebrum. Regions were labeled with DAPI (blue) and anti-SYN (green) or anti-5-HT, anti-ChAT (red), and anti-TH (magenta). **(A)** Dorsal view of SYN-positive immunostaining in the Me, Lo, and LoP. **(B)** Ventral view of immunolabeled SYN- and ChAT-positive immunostaining in the Me, Lo, and LoP. **(C)** Tissue section displaying high TH immunostaining in the LP adjacent to the PT. Dashed line in panel **(B)** indicates cells of the SG. Scale bars = 100 μm. Me, medulla; Lo, lobula; LoP, lobula plate; LP, lateral protocerebrum; OT, optic tract; PT, protocerebral tract; SYN, synapsin; D, dorsal; V, ventral; L, lateral; M, medial.

The second optic neuropil (i.e., the medulla) was dome-shaped (Figures 1C,D, 2A). Most cell bodies (cell cluster 2) associated with the medulla were located above the neuropil. Although the third optic neuropil (i.e., the lobula) was also dome-shaped, it was slightly elongated along the lateromedial axis (Figures 1C,D, 2A,B). Moreover, cell bodies in cluster 3 were visible in the vicinity of the lobula (Figures 1C,D, 2C). Consistent with other representatives of anomuran crustaceans (Krieger et al., 2010, 2012), the lobula plate was located next to the lobula and is a small neuropil that displayed a high number of immunolabeled synapsin (Figures 1C, 2A,B). Additionally, the neurohemal SG was located at the level of the lobula and bordered cell cluster 3 (Figures 1C,D, 2B). Our observation regarding the position of the lobula plate near the lobula and immediately beneath the SG in *P. camtschaticus* agreed with that in the crab *Chasmagnathus granulatus* (Sztarker et al., 2005).

The lateral protocerebrum that comprised distinct neuropils, including the terminal medulla and the hemiellipsoid body, was located proximal to the lobula (Figures 1C,D, 2C). In fact, the lateral protocerebrum exhibited significant immunostaining of synapsin; however, no clear separation between the terminal medulla and the hemiellipsoid body was detected (Figures 1C,D). The lateral protocerebrum is a part of the brain that connects with the optic neuropils *via* the optic tract (Figure 2C) and with the anterior medial protocerebral neuropil and other areas of the brain *via* the protocerebral tract.

### Distribution of Serotonin, Tyrosine Hydroxylase, and Choline Acetyltransferase in the Eyestalk

We detected 5-HT, TH, and ChAT in a majority of eyestalk regions, including the optic neuropils, SG, and lateral protocerebrum (Figures 3–11).

**FIGURE 3:**
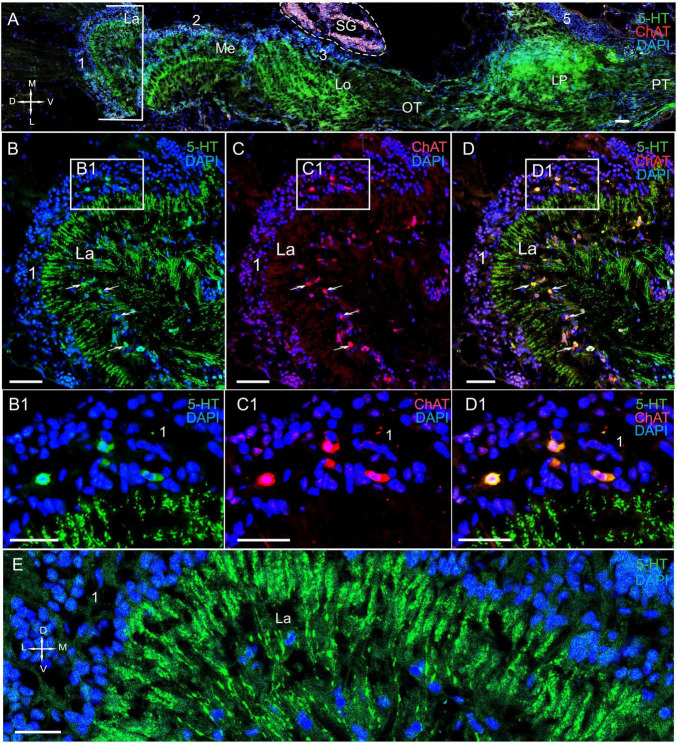
Immunolocalization of 5-HT and ChAT in the lamina of *Paralithodes camtschaticus*. **(A)** Horizontal section through an eyestalk showing positive immunostaining for ChAT and 5-HT in the optic neuropils, SG, and LP. **(B–D)** Detection of ChAT and 5-HT in cell cluster 1 above and below the plexiform layer of the La. **(B1–D1)** Double immunolabeling of ChAT and 5-HT in cell cluster 1 of the La. Arrows indicate the co-localization of 5-HT with ChAT in neurons. **(E)** 5-HT-positive immunostaining in varicose processes of the La. Green, 5-HT; red, ChAT; blue, DAPI. Dashed line in panels **(A)** indicates cells of the SG. Scale bars = **(A–D)** 100 μm and **(B1–E)** 50 μm. La, lamina; Me, medulla; Lo, lobula; LP, lateral protocerebrum; 1, 2, 3 cell clusters; OT, optic tract; PT, the protocerebral tract; D, dorsal; V, ventral; L, lateral; M, medial.

#### The Lamina

**FIGURE 4:**
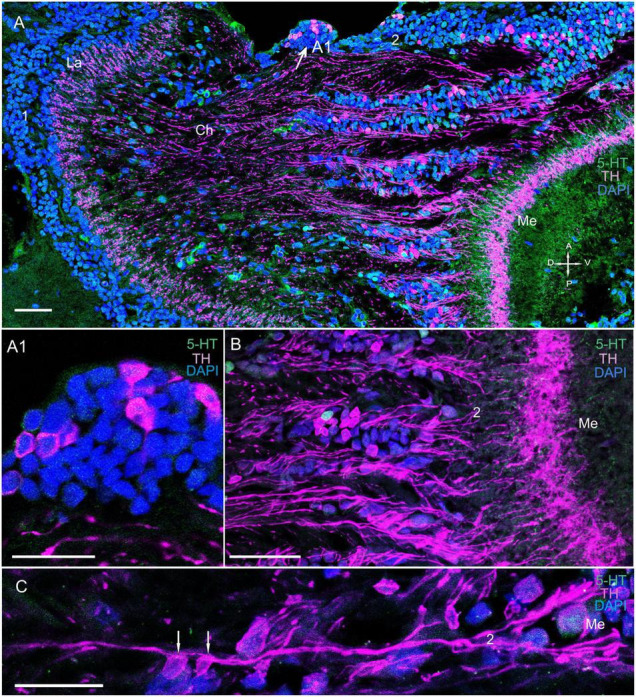
Immunolocalization of TH and 5-HT in the medulla. **(A)** TH- and 5-HT-positive bodies of neurons connecting the La to the Me. **(A1)** Localization of TH in neurons at the first Ch and closer to the Me (arrows). **(B)** Immunolocalization of TH and 5-HT in neurons and nerve fibers of the Me. **(C)** TH localization in neurons and varicose fibers connecting the La and the Me. Green, 5-HT; magenta, TH; blue, DAPI. Scale bars = **(A,B)** 100 μm and **(A1,C)** 50 μm. La, lamina; Me, medulla; 1, 2, cell clusters; Ch, optic chiasma; D, dorsal; V, ventral; A, anterior; P, posterior.

In the lamina (the first optic neuropil), few of the 5-HT- and ChAT-positive cell bodies were located in cell cluster 1 (Figures 3A–D,B1–D1). The sizes of these labeled cell bodies ranged from 15 to 20 μm and contained large nuclei that were 6–10 μm in diameter. Unfortunately, immunolabeling did not help in deciphering the processes of these cells. Notably, double immunolabeling determined that 5-HT and ChAT co-localized in the cell bodies (Figures 3B1–D1). Additionally, we identified a high number of similar-sized 5-HT- and ChAT-positive cell bodies in the proximal cell layer (below the lamina synaptic layer; Figures 3B–D and Supplementary Figure 1B). In fact, we observed co-localization of 5-HT and ChAT in most of these cell bodies. Furthermore, numerous 5-HT-positive processes with varicosities were located in the lamina plexiform layer adjacent to the first optic chiasma (Figures 3A,B,D,E). Moreover, we found numerous TH-positive fibers that interconnected the lamina and the medulla (Figures 4A–C and Supplementary Figures 1A,B, 2A–C). These fibers displayed varicosities and displayed highly intense immunostaining of TH in the lamina plexiform layer and in the first (or the outer) optic chiasma (Figure 4A and Supplementary Figures 1A,B, 2B).

#### The Medulla

**FIGURE 5:**
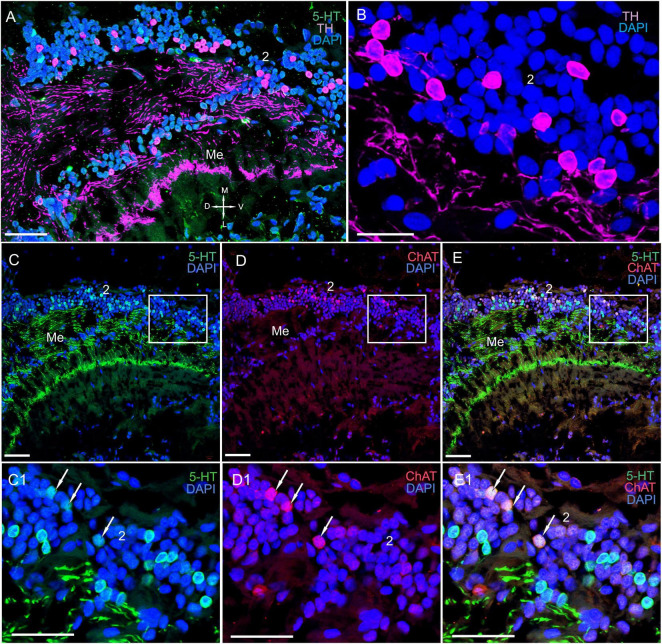
Immunolocalization of 5-HT, TH, and ChAT in the medulla. **(A)** TH- and 5-HT-positive neurons and nerve fibers in the Me. **(B)** Immunohistochemical localization of TH in neurons of cell cluster 2. **(C–E)** Double immunolabeling of 5-HT and ChAT in neurons and nerve fibers of the Me. **(C1–E1)** Higher magnification of 5-HT- and ChAT-positive neurons of the Me. Arrows indicate colocalization of 5-HT with ChAT in some of the neurons. Green, 5-HT; red, ChAT; magenta, TH; blue, DAPI. Scale bars = **(A,C–E1)** 100 μm and **(B)** 50 μm. Me, medulla; 2, cell clusters; D, dorsal; V, ventral; L, lateral; M, medial.

**FIGURE 6:**
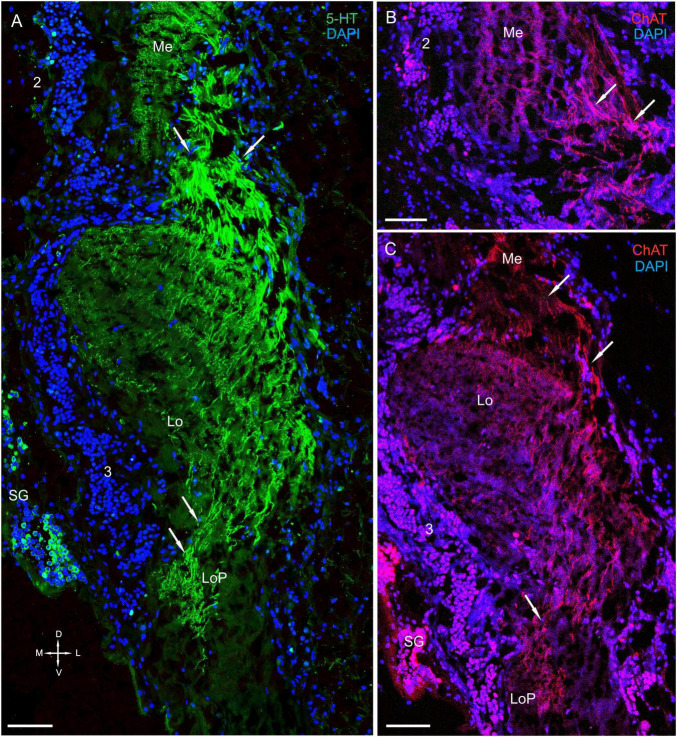
Immunolocalization of 5-HT and ChAT in the lobula and the lobula plate. **(A)** 5-HT-positive nerve fibers in the Lo and LoP. **(B)** ChAT-positive nerve fibers between in the Me and Lo. **(C)** ChAT-positive nerve fibers in the Lo and LoP. Arrows indicate immunoreactive nerve fibers. Green, 5-HT; red, ChAT; blue, DAPI. Scale bars = 100 μm. Me, medulla; Lo, lobula; 2, 3 cell clusters; LoP, lobula plate; D, dorsal; V, ventral; L, lateral; M, medial.

We detected few associations between certain TH-positive fibers and TH-positive cell bodies of cluster 2 (Figures 4A–C and Supplementary Figure 2B). These cell bodies had sizes ranging from 12 to 18 μm (Figures 4A–C, 5A,B). Interestingly, the identified TH-positive neuronal populations included a small group of TH-positive neurons that had somata that were located in the first optic chiasma (arrows in Figures 4A,A1; large arrows in Supplementary Figure 1B). Moreover, fibers that showed intense TH immunolabeling were detected in the distal portion of the medulla in layers 1 through 4 (Figures 4A, 5A and Supplementary Figures 2A–C), which comprise ∼27 to ∼34% of the depth of a neuropil that they cover (Sztarker and Tomsic, 2014). Layers 5 through 11, except for single fine processes, hardly exhibited any TH-positive immunostaining (Supplementary Figures 2B,C). Furthermore, we detected 5-HT- and ChAT-positive cell bodies in cluster 2 (Figures 3A, 5C–E); however, the TH-positive cells did not co-localize with either 5-HT- or ChAT-positive cells. Remarkably, double immunolabeling revealed that ChAT co-localized with 5-HT in most but not all 5-HT-positive perikarya (Figures 5C–E, arrowheads in C1–E1). Notably, the medulla was innervated by 5-HT-positive fibers. Layers 1 through 3 contained thin fibers diffusely projecting along the columns of the medulla and also contained labeled tangentially oriented fibers (Figures 5C,E). Thus, the medulla comprised 5-HT- and ChAT-positive fibers that formed large bundles of fibers and extended into the lobula and lobula plate (Figures 6A–C).

#### The Lobula and Lobula Plate

**FIGURE 7:**
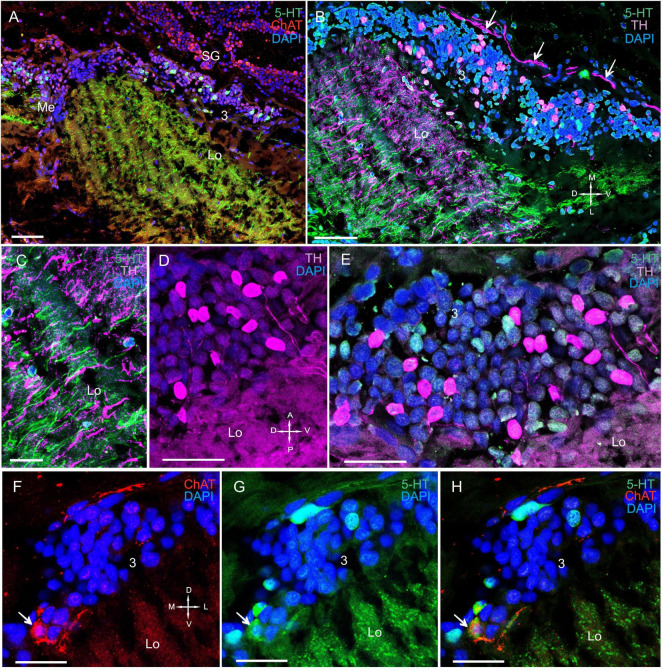
Immunolocalization of 5-HT, TH, and ChAT in the lobula. **(A)** 5-HT- and ChAT-positive cell bodies and nerve fibers in the Lo. **(B)** TH- and 5-HT-positive cell bodies and nerve fibers in the Lo with TH-positive fibers running above cell cluster 3. **(C)** TH-and 5-HT-positive fibers of the Lo. **(D)** Immunohistochemical localization of TH in the somata of cell cluster 3. **(E)** Immunohistochemical localization of 5-HT- and TH-positive neurons of cell cluster 3. **(F–H)** Double immunolabeling of ChAT and 5-HT in cell cluster 3 of the distal part of the Lo. Arrows indicate co-localization of 5-HT with ChAT in neurons. Green, 5-HT; red, ChAT; magenta, TH; blue, DAPI. Scale bars = **(A–C)** 100 μm and **(D–H)** 50 μm.

**FIGURE 8:**
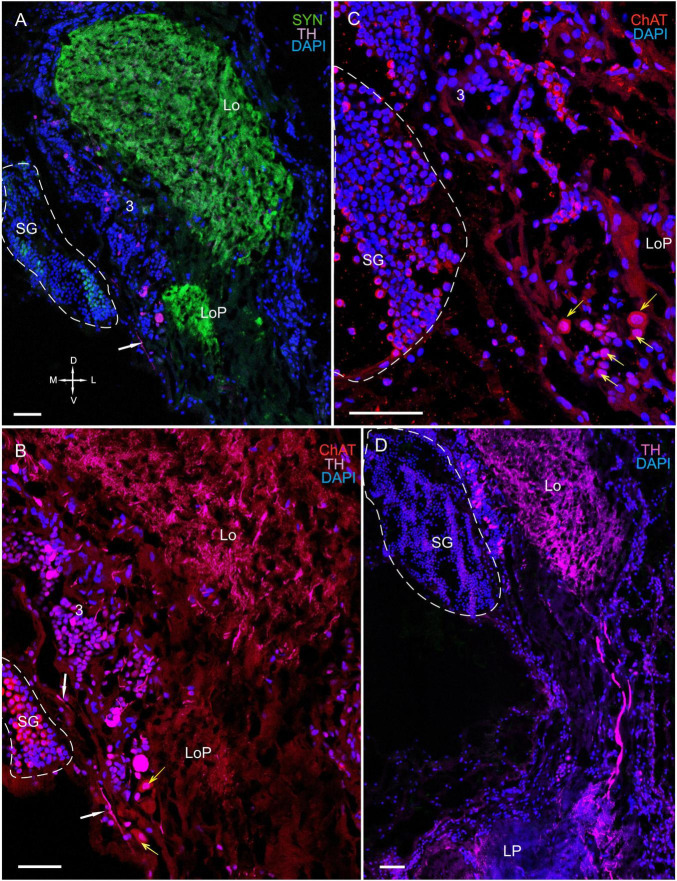
Immunolocalization of TH, ChAT, and SYN in the lobula, lobula plate, and SG. **(A)** Photographs demonstrating the position of TH-positive cell bodies near the Lo and LoP. **(B)** TH- and ChAT-positive immunostaining in the SG (yellow arrows: ChAT-positive somata). TH-positive fibers running between neurons of cluster 3 and the SG (white arrows). **(C)** ChAT-positive neurons localized near the front edge of the Lo and near the LoP. **(D)** TH-positive nerve fibers connect the Lo and LP. Green, SYN; magenta, TH; red, ChAT; blue, DAPI. Dashed lines indicate cells of the SG. Scale bars = 100 μm. Lo, lobula; LoP, lobula plate; LP, lateral protocerebrum; SYN, synapsin; D, dorsal; V, ventral; L, lateral; M, medial; A, anterior; P, posterior.

In the lobula, we detected TH, 5-HT, and ChAT in cell bodies and fibers (Figures 6A–C, 7A–H and Supplementary Figure 2A). Additionally, the labeled bodies of neurons that formed cluster 3 ranged from 10 to 18 μm (Figures 7A,B,D–H). Notably, double immunolabeling indicated that a portion of the ChAT-positive neuronal population also displayed co-localization of ChAT with 5-HT (Figures 7F–H). The anterior edge of the lobula near the lobula plate contained solitary TH-positive cell bodies ranging from 25 to 30 μm in size (Figures 8A,B) and grouped ChAT-positive neuronal perikarya ranging from 10 to 28 μm in size (Figures 8B,C).

All layers of the lobula displayed immunostaining (Figures 6A–C, 7A–C and Supplementary Figure 2A). For example, the proximal regions of layers 6 through 11, which are supplied by fibers extending from the medulla, showed highly intense 5-HT staining. By contrast, the distal region of layers 1 through 5 exhibited moderately intense 5-HT staining (Figure 6A). The lobula plate of *P. camtschaticus* received a thick bundle of 5-HT- and ChAT-positive fibers corresponding to the columns of the medulla (Figures 6A,C), and the lobula was connected to the medulla and lobula plate *via* a bundle of 5-HT- (Figure 6A) and ChAT-positive fibers (Figures 6B,C), which ran through the lobula to the lobula plate. Moreover, the lobula was invaded by thick 5-HT-positive axons from the central brain and that ramified in the neuropil (Figure 8D).

#### The Sinus Gland

**FIGURE 9:**
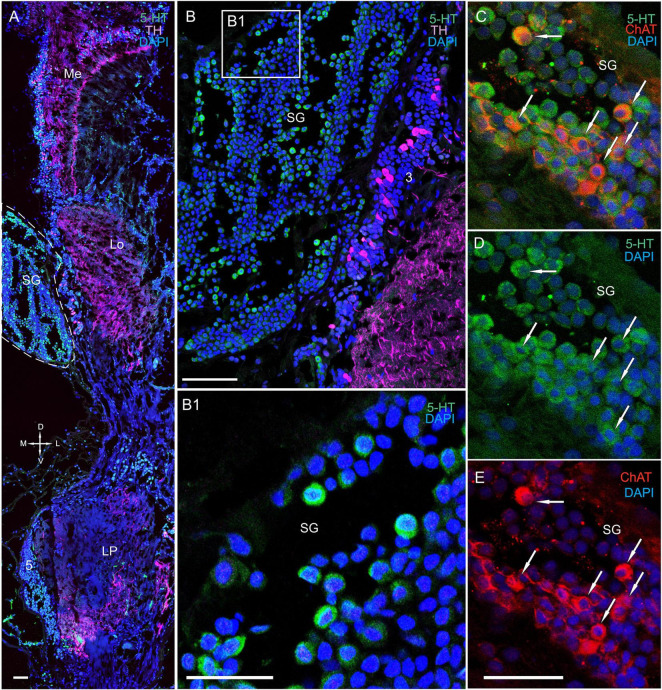
Immunolocalization of 5-HT, TH, and ChAT in the lobula and SG. **(A)** Tissue section through an eyestalk of *Paralithodes* ***camtschaticus*** displaying the location of the SG near the Lo. **(B)** Somata of TH-positive neurons located in cluster 3 near the SG. **(B1)** Inset **(B1)** shows 5-HT-positive immunostaining at higher magnification in the endogenous cells of the SG. **(C–E)** Double immunolabeling of 5-HT and ChAT in cells of the SG and showing co-localization of 5-HT with ChAT in few of the cells (arrows). Green, 5-HT; red, ChAT; magenta, TH; blue, DAPI. Dashed line indicates cells corresponding to the SG. Scale bars = **(A,B)** 100 μm and **(B1–E)** 50 μm.

The SG of *P. camtschaticus* bordered cluster 3 comprising TH-, 5– HT-, and ChAT-positive neurons (Figures 1C,D, 2B, 3A, 8D, 9A,B and Supplementary Figure 2A). Interesting, only cells ranging from 8 to 10 μm in size exhibited 5-HT- and ChAT-positive immunostaining in the SG, with these cells containing a large nucleus and a narrow rim of cytoplasm (Figures 9B,B1). Additionally, the ChAT-positive neuronal processes in few of the sections showed staining in the anterior edge of the SG (Figures 9C,E). Previous studies observed neuronal processes in the SG by electron microscopy (Hodge and Chapman, 1958). Double immunolabeling revealed that ChAT co-localized with 5-HT in few of the SG cells (Figures 9C–E). Although fibers between cluster 3 and the SG were positive for 5-HT (Figure 8B), we did not identify TH immunolabeling in SG cells (Figure 9B and Supplementary Figure 2A).

#### The Lateral Protocerebrum

**FIGURE 10:**
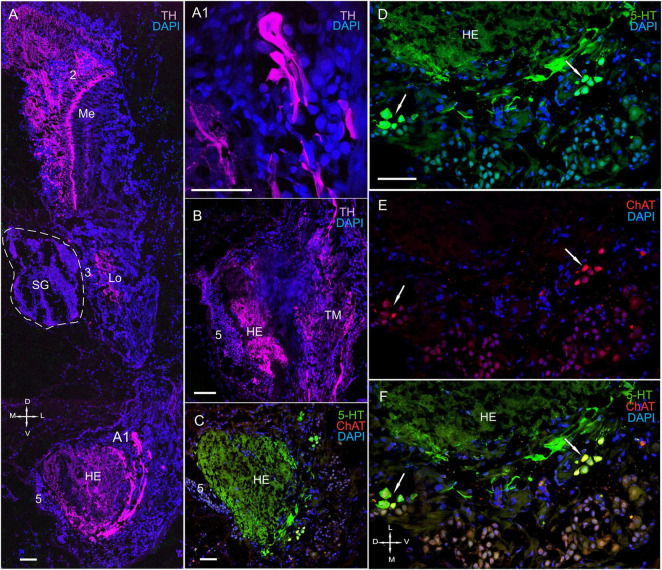
Immunolocalization of 5-HT, TH, and ChAT in the optic neuropils, SG, and lateral protocerebrum. **(A)** Tissue section showing high levels of TH-positive immunostaining in the Me, Lo, and LP. **(A1)** TH-positive cells with large varicose processes in the LP. **(B)** High levels of TH in processes of both the HE and the TM. **(C)** ChAT- and 5-HT-positive immunostaining in the HE and neurons in different regions of the TM. **(D–F)** Double immunolabeling of 5-HT and ChAT in neurons of the TM. Green, 5-HT; red, ChAT; magenta, TH; blue, DAPI. Scale bars = 100 μm. Me, medulla; Lo, lobula; HE, hemiellipsoid body; TM, terminal medulla; PT, protocerebral tract; 2, 3, 5 (ds), cell cluster; D, dorsal; V, ventral; L, lateral; M, medial; A, anterior; P, posterior.

**FIGURE 11:**
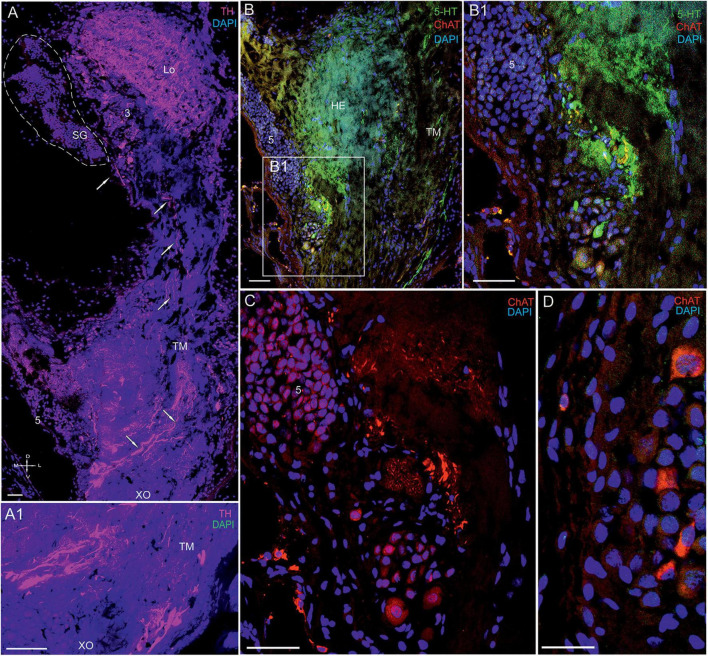
Immunolocalization of 5-HT, TH, and ChAT in the lateral protocerebrum of *Paralithodes camtschaticus*. **(A,A1)** Tissue section through the eyestalk of *P.* ***camtschaticus*** showing TH-positive immunostaining in the Lo and LP. **(A1)** Regions of the XO showing strong TH immunostaining. **(B)** TH-positive processes in the LP near the cells of cluster 5 and regions of the TM. **(B1–D)** 5-HT- and ChAT-positive neurons and processes near cell cluster 5. TH-positive immunostaining in optic neuropils and the LP. Scale bars = 100 μm. Lo, lobula; 5, cell cluster; LP, lateral protocerebrum; TM, terminal medulla; HE, the hemiellipsoid body; D, dorsal; V, ventral; L, lateral; M, medial; A, anterior; P, posterior.

Fluorescence labeling revealed the presence of TH, ChAT, and 5-HT in the lateral protocerebrum (Figures 2C, 9A, 10A–F, 11A–D and Supplementary Figure 2A); however, it was not dominated by TH-positive immunostaining. Owing to difficulties in reliable orientation of the tissue sections, we could only study the most conspicuous substructures within the lateral protocerebrum (i.e., the hemiellipsoid body and the terminal medulla) by combining anti-synapsin with anti-TH immunolabeling. We did not consider hemiellipsoid neuropils in this study. The hemiellipsoid body comprised TH-immunolabeled fibers, which branched into numerous thin fibers (Figure 10A). Moreover, cells ranging in size from 15 to 45 μm and having large varicose processes exhibited TH-positive immunostaining in the caudal part of the lateral protocerebrum (Figures 10A,A1). Indeed, their processes extended near the hemiellipsoid body and towards the optic tract (Figure 10A). The sections through the lateral protocerebrum, dissected at various levels, showed intense immunolabeling of rather coarse neuritis throughout the medulla terminalis (Figures 9A, 10B, 11A–B). Remarkably, some of these fibers projected to the protocerebral and optic tracts. Furthermore, small groups of neurons of various sizes (10–35 μm) that were located laterally to the hemielliposoid body in the terminal medulla displayed positive immunostaining for 5-HT and ChAT (Figures 10C–F). In fact, double immunolabeling revealed that 5-HT co-localized with ChAT in some of these neurons (Figures 10D–F).

We detected high levels of 5-HT and TH near cell cluster 5 (Figures 11A–C). The data also indicated that cells of various sizes (14–35 μm) contained ChAT (Figures 11C,D), whereas single neurons 15–25 μm in diameter contained 5-HT (Figure 11B1). Furthermore, we observed that numerous nerve fibers showing intense TH-positive immunostaining were present lateral to cluster 5 in the XO region (Figures 11A,A1). Notably, the TH-positive fibers were part of a larger bundle of fibers, some of which extended to the SG.

## Discussion

These results clearly demonstrate the presence of enzymes involved in DA, 5-HT, and ACh synthesis in the optic lobe of *P. camtschaticus*.

The lamina, which was the first optic neuropil, harbored 5-HT- and TH-positive processes that were distributed throughout the plexiform layer; however, we observed no co-localization of 5-HT with TH in these fibers. Most monopolar somata were unreactive to primary antibodies used in this study. Nevertheless, some cells that were identical in size and located above and below the lamina exhibited double immunolabeling of 5-HT and ChAT. It is possible that amacrine cells with displaced cell bodies are located in the layer of monopolar cells above the lamina (Glantz et al., 2000). However, the double-immunolabeled cell bodies located below the lamina are most likely those of amacrine neurons, which would be consistent with previously described observations for several crayfish species (Nässel, 1977; Glantz et al., 2000; Polanska et al., 2007) and the crab *C. granulatus* (Sztarker et al., 2009). There is evidence that neuroactive substances are possibly released by amacrine cells under quantitatively varying levels of excitation (Marder et al., 1995). These neuromodulatory substances may increase the photoreceptor sensitivity to dark adaptation and/or circadian rhythms (Aréchiga et al., 1990). Notably, 5-HT is involved in circadian clock regulation (Ichikawa, 1994; Pyza and Meinertzhagen, 1996; Chen et al., 1999) and has been identified in eyestalks of all previously studied crustacean species (Beltz and Kravitz, 1983; Elofsson, 1983; Nassel et al., 1985; Sandeman et al., 1988). Additionally, 5-HT reportedly increases the receptor potential by modulating its K^+^ conductance in various arthropod species (Aréchiga et al., 1990; Weckström, 1994; Hevers and Hardie, 1995), and another study demonstrated 5-HT as a local modulator of retinal activity (Aréchiga et al., 1990). Moreover, in insects, voltage-dependent K+ conductivities in photoreceptors are activated during depolarization, thereby reducing membrane resistance, adjusting the bandwidth in accordance with functional requirements, and causing shifts in photoreceptor performance toward higher contrast gains and lower membrane bandwidths (Heras et al., 2018).

In this study, fibers in the lamina originated from TH-positive cell bodies at the dorsal border of the medulla. These cells in *P. camtschaticus* can be identified as tangential cells, with their identification based on the location of their cell bodies at the distal edge of the medulla. Their location is also dependent on the presence of lateral processes in the lamina extending over several cartridges and an axon connecting the lamina with the medulla (Nässel, 1977; Wang-Bennett and Glantz, 1987a,b). The morphology of tangential cells has been described in detail for several crustacean species using Golgi-impregnation techniques (Nässel, 1977; Strausfeld and Nässel, 1981; Wang-Bennett and Glantz, 1987a,b; Sztarker et al., 2005, 2009). In *P. camtschaticus*, the TH-positive tangential cells exhibited few similarities with catecholaminergic tangential neurons previously identified in *Pacifastacus leniusculus* (Elofsson et al., 1977). Remarkably, the amacrine and tangential neurons form local circuits within the lamina in crustaceans (Nässel, 1977; Stowe et al., 1977; Strausfeld and Nässel, 1981; Sztarker et al., 2005, 2009; Thoen et al., 2017) and insects (Strausfeld, 1976; Douglass and Strausfeld, 2005).

### The Medulla

We detected 5-HT, ChAT, and TH in numerous immunoreactive processes in the medulla of *P. camtschaticus*. Consistent with our findings, the source of these processes can be columnar, amacrine, and tangential neurons previously identified by neuroanatomical methods in crustaceans (Sztarker and Tomsic, 2014). Apparently, few of the 5- HT-, ChAT-, and TH-positive processes in the medulla appear to be derived from 5- HT-, ChAT-, and TH-positive columnar neurons, whose bodies localize along the distal surface of the anterior medulla. Consistent with our findings, ChAT-positive columnar neurons with cell bodies along the distal surface of the medulla were identified in crayfish (Wang-Bennett et al., 1989) and *Drosophila melanogaster* (Buchner et al., 1986). Moreover, the overall distribution of ChAT in the medulla and lobula follows similar patterns between crustaceans (Wang-Bennett and Glantz, 1986; Wang-Bennett et al., 1989) and insects (Buchner et al., 1986). Furthermore, a small number of neurons in the lobula of *P. camtschaticus* displayed TH-positive immunostaining. These neurons are derived from cell bodies present above the distal surface of the medulla and have dendrite-like processes at specific levels of the medulla and terminate in the lobula (Bengochea et al., 2018). The TH- and ChAT-positive cell bodies observed at the anterior rim of the medulla in *P. camtschaticus* may be those of tangential neurons.

### The Lobula and Lobula Plate

We identified ChAT-, TH-, and 5-HT-positive processes in the lobula and lobula plate. The ChAT-positive fibers in the lobula plate are likely derived from sets of immunoreactive cell bodies at the anterior rim of the *P. camtschaticus* medulla. Indeed, relationships between the medulla, lobula, and lobula plate have been described in several crustacean species (Harzsch and Hansson, 2008; Krieger et al., 2010, 2012). Furthermore, our observation that the 5-HT- and ChAT-positive fibers connect the lobula to the lobula plate in *P. camtschaticus* is consistent with that of studies on the crab *N. granulata* and suggests that the columnar neurons projecting from the lobula convey information toward the lobula plate (Bengochea et al., 2018).

### The Lateral Protocerebrum

Our observation of highly intense TH-positive immunostaining in neurons and nerve fibers in the lateral protocerebrum in *P. camtschaticus* has been validated in studies on *N. granulata* that reported intense dopaminergic innervation (Maza et al., 2021). Similar to previously studied crustacean species, such as *Procambarus clarkii*, *P. leniusculus*, and *Scylla olivacea* (Elofsson and Klemm, 1972; Elofsson et al., 1977; Alvarez Alvarado et al., 2005; Khornchatri et al., 2015; Sayre and Strausfeld, 2019; Strausfeld et al., 2020; Maza et al., 2021), we detected TH in the XO of *P camtschaticus*. The XO is formed by neurosecretory cells that synthesize various neuropeptides and send axons to the SG (Andrew et al., 1978; Jaros, 1978; Böcking et al., 2002; Hopkins, 2012). These neuropeptides are transported along the axons for storage in the SG, from which they are subsequently circulated directly into hemolymph (Fanjul-Moles, 2006). We identified TH-positive neurons and processes on the periphery of the XO in *P. camtschaticus*. Interestingly, we identified only individual TH-positive fibers reaching the SG, although high levels of TH in processes extending from cells of the XO to the SG have been previously found in *N. granulata* (Maza et al., 2021). These data confirm the role of DA as a neurotransmitter or neuromodulator in XO neurons (Alvarez Alvarado et al., 2005; Christie, 2011). To date, DA has been consistently reported as involved in the differential regulation of activity in neurons that synthesize CHH (Kuo et al., 1995; Sarojini et al., 1995; Zou et al., 2003; Chen et al., 2020), pigment-concentrating hormones (Rodriguez-Sosa et al., 1994; Fingerman, 1997), and the distal retinal pigment lightness-assimilating hormone (Kulkarni and Fingerman, 1986). Additionally, experimental studies on the effect of DA on CHH release show that DA-induced increases in CHH and glucose levels are absent in eyestalk-ablated animals. These results show that DA enhances CHH release into hemolymph, which in turn evokes hyperglycemic responses, and that the predominant site of DA-induced CHH release is the XO-SG complex located within the eyestalk (Zou et al., 2003).

Another neurotransmitter present in the crustacean eyestalks is 5-HT (Rodriguez-Sosa et al., 1997). In the present study, we identified 5-HT in axonal branches and several neuronal varicosities in the XO. The presence of 5-HT-positive somata and fibers proximal to the XO has been described in other crustacean species, including *P. leniusculus* (Elofsson, 1983), *Cherax destructor* (Sandeman et al., 1988), and the crayfish *P. clarkii* (Rodriguez-Sosa et al., 1997).

We identified separate TH- and 5-HT-immunopositive axons in the optic nerve of *P. camtschaticus*, suggesting that some of these axons may be efferent axons running from the protocerebrum to the XO. The presence of immunopositive axons close to the neurosecretory cells of the XO and the effect of 5-HT on the activity of XO somata, as previously described (Rodriguez-Sosa et al., 1997; Saenz et al., 1997; Basu and Kravitz, 2003), indicate that 5-HT plays a modulatory role in neurosecretion (Saenz et al., 1997; García and Aréchiga, 1998; Escamilla-Chimal et al., 2001; Harlıoğlu et al., 2020). Furthermore, 5-HT involvement in regulating the release of neuropeptides, including CHH (Basu and Kravitz, 2003; Chen et al., 2020), gonad-inhibiting hormone (Richardson et al., 1991; Sarojini et al., 1995; Fingerman, 1997; Chen et al., 2003), and red- and black-pigment-dispersing hormones (Kulkarni and Fingerman, 1986), from the SG complex has been experimentally validated (Saenz et al., 1997). Although a previous study reported the presence of 5-HT in the SG (Krieger et al., 2010, 2012) and others confirmed its regulatory role in hormone release (Cooke and Sullivan, 1982; Beltz, 1988), the endogenous sources of 5-HT synthesis in the SG remain controversial.

The SG comprises axons and axon terminals of neurosecretory cells and glial cells present in the XO (Andrew et al., 1978; Jaros, 1978; Azzouna and Rezig, 2001). Glial cells in the SG have been previously studied using electron microscopy (Hodge and Chapman, 1958; Dircksen, 1992; Azzouna and Rezig, 2001), and single secretory cells having a typical neurosecretory varicosity filled with elementary granules have been described previously in the SG of *Carcinus maenas* (May and Golding, 1983). Nevertheless, the neurochemical organization and role of glial cells and neurosecretory neurons in the SG remain poorly understood. The present study demonstrated the presence of 5-HT and ChAT in endogenous cells of the SG in *P. camtschaticus*. Recent studies discovered that nitric oxide (NO) is synthesized in the SG of the crayfish *P. clarkii* (Lee et al., 2000) and the green shore crab *C. maenas* (Pitts and Mykles, 2015). The site of NO production, storage, and release is confined to supportive tissues that contain glial cells according to Pitts and Mykles (2015), the NO produced and released by supportive tissues modulates the secretion of neuropeptides from axon terminals. In *P. camtschaticus*, endogenous cells of the SG contained enzymes for the synthesis of 5-HT and Ach. Furthermore, the lobula of *P. camtschaticus* comprised cells that expressed DA, 5-HT, and Ach, with these cells positioned near the major hemolymph sinus that allows these neurotransmitters to be released into the blood stream. Moreover, the positions of 5-HT- and ChAT-positive endogenous cells in the SG indicated that 5-HT and ChAT act as local neuromodulators. Thus, our data suggest that SG cells regulate neurosecretion *via* interactions between several neurotransmitters.

## Conclusion

In summary, we identified the distribution of neurotransmitters in the optic neuropils and XO-SG complex of the eyestalks of *P. camtschaticus.* The results indicate the presence of these neurotransmitters in immunoreactive fibers and neurons, as well as endogenous SG cells, suggesting their roles in regulating the release of neurohormones, a process that occurs in several physiological reactions that determine animal behavior. Hormone levels in crustaceans are mediated by numerous humoral and neural pathways (Hodge and Chapman, 1958; Shivers, 1976; Christie, 2011). Although further physiological analysis is required to validate the presence of 5-HT and ChAT in endogenous SG cells, the present data provide a broader understanding of the role of neurotransmitters in the regulation of neurohormone release. For example, localization of 5-HT- and ChAT-positive cells in the SG indicates that 5-HT and ChAT might be local modulators that participate in regulating the secretion of neurohormones synthesized by the XO.

## Data Availability Statement

The raw data supporting the conclusions of this article will be made available by the authors, without undue reservation.

## Ethics Statement

The field studies did not involve any endangered or rare invertebrate species. To access the marine area, no specific permissions were required, as it falls within Russian state-owned land.

## Author Contributions

EK: study concept, design, and data acquisition. VD: administrative, technical, and material support and study supervision. Both authors contributed to data analysis and interpretation, drafting of the manuscript, critical revision of the manuscript for important intellectual content, and had full access to all the data in the study and take responsibility for the integrity and accuracy of the analyzed data.

## Conflict of Interest

The authors declare that the research was conducted in the absence of any commercial or financial relationships that could be construed as a potential conflict of interest.

## Publisher’s Note

All claims expressed in this article are solely those of the authors and do not necessarily represent those of their affiliated organizations, or those of the publisher, the editors and the reviewers. Any product that may be evaluated in this article, or claim that may be made by its manufacturer, is not guaranteed or endorsed by the publisher.

## Supplementary Material

Supplementary Figure 1 Immunolocalization of serotonin (5-HT) and tyrosine hydroxylase (TH) in the optic neuropil lamina of *Paralithodes camtschaticus*. **(A)** TH-positive nerve fibers between the lamina and the medulla form the first optic chiasma. **(B)** Varicose processes of TH-immunoreactivity in the lamina of a plexiform layer and at the first chiasma. Arrows indicate 5-HT in the amacrine neurons; large arrows indicate TH-positive neurons localized at the first chiasma. Abbreviations: La, lamina; 1, cell clusters; D, dorsal; V, ventral; L, lateral; M, medial; A, anterior; P, posterior. Scale bars = 100 μm.

Supplementary Figure 2 Immunolocalization of tyrosine hydroxylase (TH) in the optic neuropils of *Paralithodes camtschaticus*. **(A)** Horizontal section showing the immunohistochemical localization of TH in the optic neuropils (HN) and lateral protocerebrum; **(B,C)** Immunohistochemical localization of TH in the neurons and nerve fibers of the medulla. Abbreviations: La, lamina; Me, medulla; Lo, lobula; SG, sinus gland; OT, optic tract; LP, the lateral protocerebrum; D, dorsal; V, ventral; L, lateral; M, medial; A, anterior; P, posterior. Scale bar = 100 μm.

## References

[B1] Allayie S. A. Ravichandran S. Bhat B. A. (2011). Hormonal regulatory role of eyestalk factors on growth of heart in mud crab. Scylla serrata. *Saudi J. Biol. Sci.* 18 283–286. 10.1016/j.sjbs.2011.02.003 23961136 PMC3730571

[B2] Alvarez Alvarado R. Porras, Villalobos M. G. Calderón, Rosete G. Rodríguez-Sosa L. (2005). Dopaminergic modulation of neurosecretory cells in the crayfish. *Cell Mol. Neurobiol*. 25 345–370. 10.1007/s10571-005-3064-9 16047546 PMC11529563

[B3] Andrew R. D. Orchard I. Saleuddin A. S. M. (1978). Structural reevaluation of the neurosecretory system in the crayfish eyestalk. *Cell Tissue Res.* 190 235–246. 10.1007/BF00218172 679257

[B4] Andrew R. D. Saleuddin A. S. M. (1978). Structure and innervation of a crustacean neurosecretory cell. *Can. J. Zool.* 56 423–430. 10.1139/z78-060

[B5] Aréchiga H. Rodriguez-Sosa L. (2002). “Distributed circadian rhythmicity in the crustacean nervous system,” in *The Crustacean Nervous System*, ed. Wiese K. (Berlin: Springer), 113–122. 10.1007/978-3-662-04843-6_8

[B6] Aréchiga H. Cortes J. L. Garcia U. Rodriguez-Sosa L. (1985). Neuroendocrine correlates of circadian rhythmicity in Crustaceans. *Am. Zool*. 25 265–274. 10.1093/icb/25.1.265 31919651

[B7] Aréchiga H. Banuelos E. Frixione E. Picones A. Rodriguez-Sosa L. (1990). Modulation of crayfish retinal sensitivity by 5-hydroxytryptamine. *J. Exp. Biol.* 150 123–143. 10.1242/jeb.150.1.123 2355208

[B8] Azzouna A. Rezig M. (2001). Ultrastructural study of the sinus gland of the shrimp *Palaemonetes mesogenitor* Sollaud, 1912. *Bull. Soc. Zool.France* 126 217–219.

[B9] Basu A. C. Kravitz E. A. (2003). Morphology and monoaminergic modulation of crustacean hyperglycemic hormone-like immunoreactive neurons in the lobster nervous system. *J. Neurocytol.* 32 253–263. 10.1023/B:NEUR.0000010084.10383.3b 14724388

[B10] Beltz B. S. (1988). “Crustacean neurohormones,” in *Invertebrate Endocrinology, Vol. 2, Endocrinology of Selected Invertebrate Types*, eds Laufer H. Downer R. G. H. (New York: Alan R. Liss), 235–258. 10.1017/cbo9780511752230.014

[B11] Beltz B. S. (1999). Distribution and functional anatomy of aminecontaining neurons in decapod crustaceans. *Microsc. Res. Tech*. 44 105–120. 10.1002/(SICI)1097-0029(19990115/01)44:2/3&lt;105::AID-JEMT5&gt;3.0.CO;2-K 10084820

[B12] Beltz B. S. Kordas K. Lee M. M. Long J. B. Benton J. L. Sandeman D. C. (2003). Ecological, evolutionary, and functional correlates of sensilla number and glomerular density in the olfactory system of decapod crustaceans. *J. Comp.Neurol.* 455 260–269. 10.1002/cne.10474 12454990

[B13] Beltz B. S. Kravitz E. A. (1983). Mapping of serotonin-like immunoreactivity in the lobster nervous system. *J. Neurosci.* 3 583–602. 10.1523/JNEUROSCI.03-03-00585.1983 6338162 PMC6564544

[B14] Bengochea M. Berón, de Astrada M. Tomsic D. Sztarker J. (2018). A crustacean lobula plate: Morphology, connections, and retinotopic organization. *J. Comp. Neurol.* 526 109–119. 10.1002/cne.24322 28884472

[B15] Birren S. J. Marder E. (2013). Neuroscience: Plasticity in the Neurotransmitter Repertoire. *Science* 340 436–437. 10.1126/science.1238518 23620040

[B16] Böcking D. Dircksen H. Keller R. (2002). “The Crustacean Neuropeptides of the CHH/MIH/GI Family: Structures and Biological Activities,” in *The Crustacean Nervous System*, ed. Wiese K. (Berlin: Springer), 84–97. 10.1007/978-3-662-04843-6_6

[B17] Buchner E. Buchner S. Crawford G. Mason W. T. Salvaterra P. M. Sattelle D. B. (1986). Choline acetyltransferase-like immunoreactivity in the brain of Drosophila melanogaster. *Cell Tissue Res.* 246 57–62.

[B18] Cheng K. Y. Frye M. A. (2020). Neuromodulation of insect motion vision. *J. Comp. Physiol. A. Neuroethol. Sens Neural. Behav. Physiol.* 206 125–137. 10.1007/s00359-019-01383-9 31811398

[B19] Chen B. Meinertzhagen I. A. Shaw S. R. (1999). Circadian rhythms in light-evoked responses of the fly’s compound eye, and the effects of neuromodulators 5-HT and the peptide PDF. *J. Comp. Physiol. A.* 185 393–404. 10.1007/s003590050400 10573867

[B20] Chen H. Y. Toullec J. Y. Lee C. Y. (2020). The Crustacean Hyperglycemic Hormone Superfamily: Progress Made in the Past Decade. *Front. Endocrinol.* 11:578958. 10.3389/fendo.2020.578958 33117290 PMC7560641

[B21] Chen Y. N. Fan H. F. Hsieh S. L. Kuo C. M. (2003). Physiological involvement of DA in ovarian development of the freshwater giant prawn, *Macrobrachium rosenbergii*. *Aquaculture* 228, 383–395.

[B22] Christie A. E. (2011). Crustacean neuroendocrine systems and their signaling agents. *Cell Tissue Res.* 345 41–67. 10.1007/s00441-011-1183-9 21597913

[B23] Chung J. S. Zmora N. Katayama H. Tsutsui N. (2010). Crustacean hyperglycemic hormone (CHH) neuropeptidesfamily: functions, titer, and binding to target tissues. *Gen. Comp. Endocrinol.* 166 447–454. 10.1016/j.ygcen.2009.12.011 20026335

[B24] Cooke I. M. Sullivan R. E. (1982). “Hormones and neurosecretion,” in *The Biology of Crustacea, Vol. 3. Neurobiology: Structure and Function*, eds Atwood H. L. Sandeman D. C. (New York: Academic Press) 206–278.

[B25] Cournil I. Helluy S. M. Beltz B. S. (1994). Dopamine in the lobster *Homarus gammarus*. I. Comparative analysis of dopamine and tyrosine hydroxylase immunoreactivities in the nervous system of the juvenile. *J. Comp. Neurol.* 344 455–469. 10.1002/cne.903440308 7914897

[B26] Crow T. Bridge M. S. (1985). Serotonin modulates photoresponses in *Hermissenda* type-B photoreceptors. *Neurosci. Lett.* 60 83–88. 10.1016/0304-3940(85)90385-4 2997674

[B27] De Kleijn D. P. Van Herp F. (1995). Molecular biology of neurohormone precursors in the eyestalk of Crustacea. *Comp. Biochem. Physiol. B, Biochemistry and Molecular Biology.* 112 573–579. 10.1016/0305-0491(95)00126-3 8590372

[B28] Dircksen H. (1992). Fine structure of the neurohemal sinus gland of the shore crab, *Carcinus maenas*, and immuno-electron-microscopic identificationof neurosecretory endings according to their neuropeptide contents. *Cell Tissue Res*. 269 249–266. 10.1007/BF00319616 1423493

[B29] Donaldson W. E. Byersdorfer S. C. (2005). *Biological Field Techniques for Lithodid Crabs.* Alaska: University of Alaska, 82.

[B30] Douglass J. K. Strausfeld N. J. (2005). Sign-conserving amacrine neurons in the fly’s external plexiform layer. *Vis. Neurosci.* 22 345–358. 10.1017/S095252380522309X 16079009

[B31] Dvoretsky A. G. Dvoretsky V. G. (2013). Population dynamics of the invasive lithodid crab, *Paralithodes camtschaticus*, in a typical bay of the Barents Sea. *ICES J. Mar. Sci.* 70 1255–1262. 10.1093/icesjms/fst037

[B32] Dvoretsky A. G. Dvoretsky V. G. (2015). Commercial fish and shellfish in the Barents Sea: Have introduced crab species affected the population trajectories of commercial fish? *Rev. Fish Biol. Fish*. 25 297–322. 10.1007/s11160-015-9382-1

[B33] Dvoretsky A. G. Dvoretsky V. G. (2018). Red king crab (*Paralithodes camtschaticus*) fisheries in Russian waters: historical review and present status. *Rev. Fish Biol. Fish.* 28 331–353. 10.1007/s11160-017-9510-1

[B34] Dvoretsky A. G. Tipisova E. V. Elfimova A. E. Alikina V. A. Dvoretsky V. G. (2021). Sex hormones in hemolymph of red king crabs from the Barents Sea. *Animals* 11:2149. 10.3390/ani11072149 34359277 PMC8300720

[B35] Dyachuk V. A. Maiorova M. A. Odintsova N. A. (2015). Identification of β integrin-like- and fibronectin-like proteins in the bivalve mollusk *Mytilus trossulus*. *Dev. Growth. Differ.* 57 515–528. 10.1111/dgd.12234 26183371

[B36] Elofsson R. Dahl E. (1970). The optic neuropiles and chiasmata of Crustacea. *Z. Zellforsch*. 107 343–360. 10.1007/BF00336672 5448473

[B37] Elofsson R. (1983). 5-HT immunoreactivity in the central nervous system of the crayfish. *Pacifastacus leniusculus*. *Cell Tissue Res*. 232 221–236. 10.1007/BF00222385 6349819

[B38] Elofsson R. Laxmyr L. Rosengren E. Hansson C. (1982). Identification and quantitative measurements of biogenic amines and dopa in the central nervous system and haemolymph of the crayfish, *Pacifastacus leniusculus*. *Comp. Biochem. Physiol.* 71 195–201. 10.1016/0306-4492(82)90036-3

[B39] Elofsson R. Klemm N. (1972). Monoamine containing neurons in the optic ganglion of crustacean and insects. *Z. Zellforsch. Mikrosk. Anat.* 133 475–479. 10.1007/BF00307130 5082398

[B40] Elofsson R. Nässel D. Myhrberg H. (1977). A catecholaminergic neuron connecting the first two optic neuropiles (lamina ganglionaris and medulla externa) of the crayfish *Pacifastaeus leniusculus*. *Cell Tissue Res.* 182 287–297. 10.1007/BF00219765 336211

[B41] Escamilla-Chimal E. G. Van Herp F. Fanjul-Moles M. L. (2001). Daily variations in crustacean hyperglycemic hormone and serotonin immunoreactivity during the development of crayfish. *J. Exp. Biol.* 204 1073–1081. 10.1242/jeb.204.6.1073 11222126

[B42] Fanjul-Moles M. L. (2006). Biochemical and functional aspects of crustacean hyperglycemic hormone in decapod crustaceans: review and update. *Comp. Biochem. Physiol. C Toxicol. Pharmacol.* 142 390–400. 10.1016/j.cbpc.2005.11.021 16403679

[B43] Fingerman M. (1992). “Decapod crustacean glands,” in *Microscopic Anatomy of Invertebrates, Vol. 10. Decapod Crustacea*, eds Harrison F. W. Humes A. G. (New York: Wiley-Liss), 345–394.

[B44] Fingerman M. (1997). Roles of neurotransmitters in regulating reproductive hormone release and gonadal maturation in decapods crustaceans. *Invert. Reprod. Develop*. 31 47–54. 10.1080/07924259.1997.9672562

[B45] Fingeman M. Nagabushanam R. (1992). Control of the release of crustzcean hormones by neuroregdators. *Comp. Binchem. Physiol.* 102C 343–352.

[B46] García U. Aréchiga H. (1998). Regulation of crustacean neurosecretory cell activity. *Cell. Mol. Neurobiol.* 18 81–99. 10.1023/a:1022527210808 9524731 PMC11560232

[B47] Glantz R. M. Miller C. S. Nässel D. R. (2000). Tachykinin-related peptideand GABA-mediated presynaptic inhibition in crayfish photoreceptors. *J. Neurosci.* 20 1780–1790. 10.1523/JNEUROSCI.20-05-01780.2000 10684879 PMC6772939

[B48] Glantz R. M. Kirk M. D. Aréchiga H. (1983). Light input to crustacean neurosecretory cells. *Brain Res.* 265 307–311. 10.1016/0006-8993(83)90347-5 6850335

[B49] Harlıoğlu M. M. Farhadi A. Harlıoğlu A. G. (2020). Roles of Neurotransmitters in Decapod Reproduction. *Thalassas.* 36 633–639. 10.1007/s41208-020-00202-2

[B50] Harzsch S. Anger K. Dawirs R. R. (1997). Immunocytochemical detection of acetylated alpha-tubulin and *Drosophila* synapsin in the embryonic crustacean nervous system. *Int. J. Dev. Biol.* 41 477–484. 9240564

[B51] Harzsch S. Hansson B. S. (2008). Brain architecture in the terrestrial hermit crab *Coenobita clypeatus* (Anomura, Coenobitidae), a crustacean with a goodaerial sense of smell. *BMC Neurosci*. 9:58. 10.1186/1471-2202-9-58 18590553 PMC2459186

[B52] Harzsch S. Waloszek D. (2000). Serotonin-immunoreactive neurons in the ventral nerve cord of Crustacea: a character to study aspects of arthropod phylogeny. *Arthropod. Struct. Dev.* 29 307–322. 10.1016/s1467-8039(01)00015-9 18088936

[B53] Hemmi J. M. (2005). Predator avoidance in fiddler crabs: 2. The visual cues. *Animal. Behaviour*. 69 615–625. 10.1016/j.anbehav.2004.06.019

[B54] Hemmi J. M. Tomsic D. (2012). The neuroethology of escape in crabs: From sensory ecology to neurons and back. *Curr. Opin. Neurobiol.* 22 194–200. 10.1016/j.conb.2011.11.012 22176799

[B55] Heras F. J. H. Vähäsöyrinki M. Niven J. E. (2018). Modulation of voltage-dependent K^+^ conductances in photoreceptors trades off investment in contrast gain for bandwidth. *PLoS Comput. Biol.* 14:e1006566. 10.1371/journal.pcbi.1006566 30399147 PMC6239345

[B56] Hevers W. Hardie R. C. (1995). Serotonin modulates the voltage dependence of delayed rectifier and Shaker potassium channels in *Drosophila* photoreceptors. *Neuron* 14 845–856. 10.1016/0896-6273(95)90228-7 7718246

[B57] Hildebrand J. G. Townsel J. G. Kravitz E. A. (1974). Distribution of acetylcholine, choline, choline acetyltransferase and acetylcholinesterase in regions and single identified axons of the lobster nervous system. *J. Neurochem.* 23 951–963. 10.1111/j.1471-4159.1974.tb10747.x 4215870

[B58] Hodge M. H. Chapman G. B. (1958). Some observations on the fine structure of the sinus gland of a land crab. *Gecarcinus lateralis*. *J. Biophys. Biochem. Cytol.* 4 571–574. 10.1083/jcb.4.5.571 13587551 PMC2224553

[B59] Hopkins P. M. (2012). The eyes have it: A brief history of crustacean neuroendocrinology. *Gen. Comp. Endocrinol.* 175 357–366. 10.1016/j.ygcen.2011.12.002 22197211

[B60] Ito K. Shinomiya K. Ito M. Armstrong J. D. Boyan G. Hartenstein V. (2014). Insect Brain Name Working Group. A systematic nomenclature for the insect brain. *Neuron* 81 755–765. 24559671 10.1016/j.neuron.2013.12.017

[B61] Itoh N. Slemmon J. R. Hawke D. H. Williamson R. Morita E. Itakura K. (1986). Cloning of Drosophila choline acetyltransferase cDNA. *Proc. Natl. Acad. Sci. U.S.A.* 83 4081–4085. 10.1073/pnas.83.11.4081 3086876 PMC323670

[B62] Ichikawa T. (1994). Light suppresses the activity of serotonin-immunoreactive neurons in the optic lobe of the swallowtail butterfly. *Neurosci. Lett*. 172 115–118. 10.1016/0304-3940(94)90675-0 8084513

[B63] Jaros P. P. (1978). Tracing of neurosecretory neurons in crayfish optic ganglia by cobalt iontophoresis. *Cell Tissue Res.* 194 297–302. 10.1007/BF00220396 728967

[B64] Khornchatri K. Kornthong N. Saetan J. Tinikul Y. Chotwiwatthanakun C. Cummins S. F. (2015). Distribution of serotonin and dopamine in the central nervous system of the female mud crab, *Scylla olivacea* (Herbst). *Acta Histochem.* 117 196–204. 10.1016/j.acthis.2014.12.006 25618422

[B65] Klappenbach M. Maldonado H. Locatelli F. Kaczer L. (2012). Opposite actions of dopamine on aversive and appetitive memories in the crab. *Learn. Mem*. 19 73–83. 10.1101/lm.024430.111 22267303

[B66] Kotsyuba E. Dyachuk V. (2021). Localization of neurons expressing choline acetyltransferase, serotonin and/or FMRFamide in the central nervous system of the decapod shore crab *Hemigrapsus sanguineus*. *Cell Tissue Res.* 383 959–977. 10.1007/s00441-020-03309-3 33237479

[B67] Kloppenburg P. Erber J. (1995). The modulatory effects of serotonin and octopamine in the visual system of the honey bee (*Apis mellifera* L.) II. Electrophysiological analysis of motion-sensitive neurons in the lobula. *J. Comp. Physiol.* 176 119–129.

[B68] Krieger J. Sandeman R. E. Sandeman D. C. Hansson B. S. Harzsch S. (2010). Brain architecture of the largest living land arthropod, the Giant Robber Crab *Birgus latro* (Crustacea, Anomura, Coenobitidae): evidence for a prominent central olfactory pathway? *Front. Zool.* 7:25. 10.1186/1742-9994-7-25 20831795 PMC2945339

[B69] Krieger J. Sombke A. Seefluth F. Kenning M. Hansson B. Harzsch S. (2012). Comparative brain architecture of the European shore crab *Carcinus maenas* (Brachyura), the common hermit crab *Pagurus bernhardus* (Anomura) with notes on other marine hermit crabs. *Cell Tissue Res.* 348 47–69. 10.1007/s00441-012-1353-4 22374330

[B70] Krieger J. Braun P. Rivera N. T. Schubart C. D. Müller C. H. G. Harzsch S. (2015). Comparative analyses of olfactory systems in terrestrial crabs (Brachyura): evidence for aerial olfaction? *Peer J.* 3:e1433. 10.7717/peerj.1433 26713228 PMC4690415

[B71] Kulkarni G. K. Fingerman M. (1986). Distal retinal pigment of the fiddler crab, Uca pugilator: evidence for stimulation of release of light adapting and dark-adapting hormones by neurotransmitters. *Comp. Binchem. Physiol. C*. 84 219–224. 10.1016/0742-8413(86)90086-1

[B72] Kuo C. M. Hsu C. R. Lin C. Y. (1995). Hyperglycaemic effects of dopamine in tiger shrimp, *Penaeus monodon*. *Aquaculture* 135, 161–172.

[B73] Lee C. Y. Zou H. S. Yau S. M. Ju Y. R. Liau C. S. (2000). Nitric oxide synthase activity and immunoreactivity in the crayfish *Procambarus clarkia*. *Neuroreport* 11 1273–1276. 10.1097/00001756-200004270-00026 10817606

[B74] Mancillas J. R. McGinty J. F. Selverston A. I. Karten H. Bloom F. E. (1981). Immunocytochemical localization of enkephalin and substance P in retina and eyestalk neurones of lobster. *Nature* 293 576–578. 10.1038/293576a0 6169995

[B75] Mangerich S. Keller R. (1988). Localization of pigment-dispersing hormone (PDH) immunoreactivity in the central nervous system of *Carcinus maenas* and *Orconectes limosus* (Crustacea), with reference to FMRFamide immunoreactivity in O. limosus. *Cell Tissue Res.* 253 199–208. 10.1007/BF00221755 3416337

[B76] Marder E. Christie A. E. Kilman V. L. (1995). Functional organization of cotransmission systems: lessons from small nervous systems. *Invert. Neurosci.* 1 105–112. 10.1007/BF02331908 9372135

[B77] Marder E. Thirumalai V. (2002). Cellular, synaptic and network effects of neuromodulation. *Neural. Netw.* 15 479–493. 10.1016/s0893-6080(02)00043-6 12371506

[B78] May B. A. Golding D. W. (1983). Aspects of secretory phenomena within the sinus gland of *Carcinus maenas* L. An ultrastructural study. *Cell Tissue Res.* 228 245–254. 10.1007/BF00204876 6825164

[B79] Maza F. J. Sztarker J. Shkedy A. Peszano V. N. Locatelli F. F. Delorenzi A. (2016). Context-dependent memory traces in the crab’s mushroom bodies: Functional support for a common origin of high-order memory centers. *Proc. Natl. Acad. Sci.U.S.A.* 113 E7957–E7965. 10.1073/pnas.1612418113 27856766 PMC5150377

[B80] Maza F. J. Sztarker J. Cozzarin M. E. Lepore M. G. Delorenzi A. (2021). A crabs’ high-order brain center resolved as a mushroom body-like structure. *J. Comp. Neurol.* 529 501–523. 10.1002/cne.24960 32484921

[B81] Medan V. Berón, de Astrada M. Scarano F. Tomsic D. (2015). A network of visual motion-sensitive neurons for computing object position in an arthropod. *J. Neurosci*. 17 6654–6666. 10.1523/JNEUROSCI.4667-14.2015 25926445 PMC6605188

[B82] Murai M. Backwell P. R. Y. (2006). A conspicuous courtship signal in the fiddler crab *Uca perplexa*: Female choice based on display structure. *Behav. Ecol. Sociobiol.* 60 736–741. 10.1007/s00265-006-0217-x

[B83] Nässel D. R. (1977). Types and arrangement of neurons in the crayfish optic lamina. *Cell Tissue Res.* 179 45–75. 10.1007/BF00278462 870207

[B84] Nassel D. R. Meyer E. R. Klemm N. (1985). Mapping and ultrastructure of serotonin-immunoreactive neurons in the optic lobes of three insect species. *J. Comp. Neurol.* 232 190–204. 10.1002/cne.902320205 3973090

[B85] Nusbaum M. P. Blitz D. M. Swensen A. M. Wood D. Marder E. (2001). The roles of co-transmission in neural network modulation. *Trends Neurosci.* 24 146–154. 10.1016/s0166-2236(00)01723-9 11182454

[B86] Pavlova L. V. Britayev T. A. Rzhavsky A. V. (2007). Benthos elimination by juvenile red king crabs *Paralithodes camtschaticus* (Tilesius, 1815) in the Barents Sea coastal zone: Experimental data. *Dokl. Biol. Sci.* 414 231–234. 10.1134/S0012496607030180 17668630

[B87] Pérez-Polanco P. Garduño J. Cebada J. Zarco N. Segovia J. Lamas M. (2011). GABA and GAD expression in the X-organ sinus gland system of the *Procambarus clarkii* crayfish: inhibition mediated by GABA between X-organ neurons. *J. Comp. Physiol.* 197 923–938. 10.1007/s00359-011-0653-6 21626307

[B88] Pitts N. L. Mykles D. L. (2015). Nitric oxide production and sequestration in the sinus gland of the green shore crab *Carcinus maenas*. *J. Exp. Biol.* 218 353–362. 10.1242/jeb.113522 25452501

[B89] Polanska M. A. Yasuda A. Harzsch S. (2007). Immunolocalisation of crustacean-SIFamide in the median brain and eyestalk neuropils of the marbled crayfish. *Cell Tissue Res.* 330 331–344. 10.1007/s00441-007-0473-8 17828557

[B90] Polanska M. A. Tuchina O. Agricola H. Hansson B. S. Harzsch S. (2012). Neuropeptide complexity in the crustacean central olfactory pathway: immunolocalization of A-type allatostatins and RFamide-like peptides in the brain of a terrestrial hermit crab. *Molecular Brain*. 5:29. 10.1186/1756-6606-5-29 22967845 PMC3523048

[B91] Pyza E. Meinertzhagen I. A. (1996). Neurotransmitters regulate rhythmic size changes amongst cells in the fly’s optic lobe. *J. Comp. Physiol. A* 178 33–45. 10.1007/BF00189588 8568723

[B92] Rajendiran S. Vasudevan S. (2016). Localization and identification of crustacean hyperglycemic hormone producing neurosecretory cells in the eyestalk of blue swimmer crab. *Portunus pelagicus*. *Microsc. Res. Tech*. 79 1024–1030. 10.1002/jemt.22737 27460068

[B93] Rao K. R. (2001). Crustacean pigmentary effect hormones: chemistry and functions of RPCH, PDH, and related peptides. *Amer. Zool.* 41 364–379. 10.1093/icb/41.3.364 31919651

[B94] Richardson H. G. Deecaraman M. Fingerman M. (1991). The effect of biogenic amines on ovarian development in the fiddler crab. *Uca pugilator*. *Comp. Biochem. Physiol. C* 99 53–56. 10.1016/0742-8413(91)90074-4

[B95] Richter S. Loesel R. Purschke G. Schmidt-Rhaesa A. Scholtz G. Stach T. (2010). Invertebrate neurophylogeny - suggested terms and definitions for a neuroanatomical glossary. *Front. Zool*. 7:29. 10.1186/1742-9994-7-29 21062451 PMC2996375

[B96] Rodriguez-Sosa L. Calderón J. Becerra E. Aréchiga H. (1994). Regional distribution and immunocytological localization of red pigment concentrating hormone in the crayfish eyestalk. *Gen. Comp. Endocrinol*. 95, 443–456. 7821781 10.1006/gcen.1994.1144

[B97] Rodriguez-Sosa L. Picones A. Rosete G. Arechiga Y. S. (1997). Localization and release of 5-hydroxytryptamine in the crayfish eyestalk. *J. Exp. Biol.* 200 3067–3077. 10.1242/jeb.200.23.3067 9359895

[B98] Rudolph P. H. Spaziani E. (1990). Distribution of serotonergic neurons in the eyestalk and brain of the crab. *Cancer antennarius*. *Comp. Biochem. Physiol.* 97 241–245. 10.1016/0742-8413(90)90134-u

[B99] Saenz F. Garcia U. Arechiga U. (1997). Modulation of electrical activity by 5-hydroxytrytamine in crayfish neurosecretory cells. *J. Exp. Biol.* 200 3079–3090. 10.1242/jeb.200.23.3079 9359896

[B100] Salvaterra P. M. Kitamoto T. (2001). Drosophila cholinergic neurons and processes visualized with Gal4/UAS-GFP. *Brain Res. Gene Expr. Patterns* 1 73–82. 10.1016/s1567-133x(01)00011-4 15018821

[B101] Sandeman D. C. Sandeman R. E. Aitken A. R. (1988). Atlas of serotonin-containing neurons in the optic lobes and brain of the crayfish *Cherax destructor*. *J. Comp. Neurol.* 269 465–478. 10.1002/cne.902690402 3372724

[B102] Sandeman D. C. Scholtz G. Sandeman R. E. (1993). Brain Evolution in Decapod Crustacea. *J. Exp. Zool.* 265 112–133. 10.1002/jez.1402650204

[B103] Sandeman D. C. Sandeman R. E. Derby C. Schmidt M. (1992). Morphology of the Brain of Crayfish, Crabs, and Spiny Lobsters: A Common Nomenclature for Homologous Structures. *Biol. Bull.* 183 304–326. 10.2307/1542217 29300672

[B104] Santhoshi S. Sugumar V. Munuswamy N. (2008). Histological and immunocytochemical localization of serotonin-like immunoreactivity in the brain and optic ganglia of the Indian white shrimp, *Fenneropenaeus indicus*. *Microsc. Res. Tech.* 71 186–195. 10.1002/jemt.20511 17661386

[B105] Sarojini R. Nagabhushanam R. Devi M. Fingerman M. (1995). Dopaminergic inhibition of 5-hydroxytryptamine-stimulated testicular maturation in the fiddler crab, *Uca pugilator*. *Comp. Biochem. Physiol.* 111 287–292. 10.1016/0742-8413(95)00051-o

[B106] Sayre M. E. Strausfeld N. J. (2019). Mushroom bodies in crustaceans: Insect-like organization in the caridid shrimp *Lebbeus groenlandicus*. *J. Comp. Neurol.* 527 2371–2387. 10.1002/cne.24678 30861118

[B107] Sinakevitch I. Douglass J. K. Scholtz G. Loesel R. Strausfeld N. J. (2003). Conserved and convergent organization in the optic lobes of insects and isopods, with reference to other crustacean taxa. *J. Comp. Neurol*. 467 150–172. 10.1002/cne.10925 14595766

[B108] Siwicki K. S. Bishon C. A. (1986). Mapping of proctolin-like immunoreactivity in the nervous system of lobster and crayfish. *J. Comp. Neurol.* 243 435–453. 10.1002/cne.902430402 3512628

[B109] Schueler P. A. Madden A. J. Herman W. S. Elder R. (1986). Immunohistochemical mapping of distal retinal pigment hormone in the crayfish central nervous-system. *Sot. Neuroscl. Abstr*. 12:242.

[B110] Stevens B. G. Lovrich G. A. (2014). King crabs of the world: species and distributions. In: Stevens B.G. (ed.). *King crabs of the world: biology and fisheries management.* Boca Raton: CRC Press, 1–30.

[B111] Shivers R. R. (1976). Exocytosis of neurosecretory granules from the crustacean sinus gland in freeze-fracture. *J. Morphol.* 150 227–252. 10.1002/jmor.1051500111 30278607

[B112] Stewart M. J. Stewart P. Sroyraya M. Soonklang N. Cummins S. F. Hanna P. J. (2013). Cloning of the crustacean hyperglycemic hormone and evidence for molt-inhibiting hormone within the central nervous system of the blue crab *Portunus pelagicus*. *Comp. Biochem. Physiol. A*. 164 276–290. 10.1016/j.cbpa.2012.10.029 23103673

[B113] Stowe S. Ribi W. A. Sandeman D. C. (1977). The organisation of the lamina ganglionaris of the crabs *Scylla serrata* and *Leptograpsus variegatus*. *Cell Tissue Res.* 178 517–532. 10.1007/BF00219572 858157

[B114] Strausfeld N. J. (1976). *Atlas of an Insect Brain.* Berlin: Springer-Verlag.

[B115] Strausfeld N. J. Nässel D. R. (1981). “Neuroarchitectures serving compound eyes of Crustacea and insects,” in *Comparative Physiology and Evolution of Vision in Invertebrates. Handbook of Sensory Physiology, Vol VII/6B*, ed. Autrum H. (Berlin: Springer), 1–132. 10.1007/978-3-642-66907-1_1

[B116] Strausfeld N. J. Wolff G. H. Sayre M. E. (2020). Mushroom body evolution demonstrates homology and divergence across Pancrustacea. *eLife* 9:e52411. 10.7554/eLife.52411 32124731 PMC7054004

[B117] Strausfeld N. J. (2021). Mushroom bodies and reniform bodies coexisting in crabs cannot both be homologs of the insect mushroom body. *J. Comp. Neurol.* 12 3265–3271. 10.1002/cne.25152 33829500

[B118] Sullivan J. M. Beltz B. S. (2004). Evolutionary changes in the olfactory projection neuron pathways of eumalacostracan crustaceans. *J. Comp. Neurol*. 470 25–38. 10.1002/cne.11026 14755523

[B119] Sztarker J. Strausfeld N. J. Tomsic D. (2005). Organization of optic lobes that support motion detection in a semiterrestrial crab. *J. Comp. Neurol.* 493 396–411. 10.1002/cne.20755 16261533 PMC2638986

[B120] Sztarker J. Strausfeld N. Andrew D. Tomsic D. (2009). Neural organization of first optic neuropils in the littoral crab *Hemigrapsus oregonensis* and the semiterrestrial species *Chasmagnathus granulatus*. *J. Comp. Neurol.* 513 129–150. 10.1002/cne.21942 19123235 PMC4876859

[B121] Sztarker J. Tomsic D. (2014). Neural Organization of the second optic neuropil, the medulla, in the highly visual semiterrestrial crab *Neohelice granulata*. *J. Comp. Neurol.* 522 3177–3193. 10.1002/cne.23589 24659096

[B122] Thoen H. H. Strausfeld N. J. Marshall J. (2017). Neural organization of afferent pathways from the stomatopod compound eye. *J. Comp. Neurol*. 525, 3010–3030. 28577301 10.1002/cne.24256

[B123] Thoen H. H. Wolff G. H. Marshall J. Sayre M. E. Strausfeld N. J. (2019). The Reniform Body: An Integrative Lateral Protocerebral Neuropil Complex of Eumalacostraca identified in Stomatopoda and Brachyura. *J. Comp. Neurol.* 1:16. 10.1002/cne.24788 31621907

[B124] Tilesius G. (1815). De cancris Camtschaticis, oniscis, entomostracis et cancellis marinis microscopicis noctilucentibus, Cum tabulis IV. Aeneis et appendice adnexo de acaris et ricinis Camtschaticis. *Mémoires de l’Académie Impériale des Sciences de St. Pétersbourg* 5, 331–405, pls. 5–8.

[B125] Tomsic D. Sztarker J. Berón, de Astrada M. Oliva D. Lanza E. (2017). The predator and prey behaviors of crabs: from ecology to neural adaptations. *J. Exp. Biol.* 220 2318–2327. 10.1242/jeb.143222 28679790

[B126] Wang-Bennett L. T. Glantz R. M. (1986). Immunocytochemical visualization of acetylcholine and glutamate in the brain and eyestalk of crayfish. *Sot. Neurosci. Abstr.* 12:243.

[B127] Wang-Bennett L. T. Glantz R. M. (1987a). The functional organization of the crayfish lamina ganglionaris. I. Nonspiking monopolar cells. *J. Comp. Physiol*. 161 131–145. 10.1007/BF00609461 3612592

[B128] Wang-Bennett L. T. Glantz R. M. (1987b). The functional organization of the crayfish lamina ganglionaris. II. Large field spiking and nonspiking cells. *J. Comp. Physiol*. 161 147–160. 10.1007/BF00609462 3612593

[B129] Wang-Bennett L. T. Pfeiffer C. Arnold J. Glantz R. M. (1989). Acetylcholine in the crayfish optic lobe: concentration profile and cellular localization. *J. Neurosci*. 9 1864–1871. 10.1523/JNEUROSCI.09-06-01864.1989 2723754 PMC6569739

[B130] Webster S. G. Keller R. (1987). “Physiology and biochemistry of crustacean neurohormonal peptides,” in *Peptides and Amines in Invertebrates*, eds Thorndyke M. Goldsworthy G. J. (Cambridge: Cambridge University Press), 173–196. 10.1017/cbo9780511752230.011

[B131] Weckström M. (1994). Voltage-activated outward currents in adult and nymphal locust photoreceptors. *J. Comp. Physiol. A*. 174 795–801.

[B132] Wolff G. Harzsch S. Hansson B. S. Brown S. Strausfeld N. (2012). Neuronal organization of the hemiellipsoid body of the land hermit crab, *Coenobita clypeatus*: correspondence with the mushroom body ground pattern. *J. Comp. Neurol.* 520 2824–2846. 10.1002/cne.23059 22547177

[B133] Wood D. E. Derby C. D. (1996). Distribution of dopamine-like immunoreactivity suggests a role for dopamine in the courtship display behavior of the blue crab, *Callinectes sapidus*. *Cell Tissue Res.* 285 321–330. 10.1007/s004410050649 8766168

[B134] Yasuyama K. Salvaterra P. M. (1999). Localization of choline acetyltransferase expressing neurons in *Drosophila* nervous system. *Microsc. Res. Tech.* 45 65–79. 10.1002/(SICI)1097-0029(19990415)45:2&lt;65::AID-JEMT2&gt;3.0.CO;2-0 10332725

[B135] Zeil J. Hemmi J. M. (2006). The visual ecology of fiddler crabs. *J. Comp. Physiol. A.* 192 1–25. 10.1007/s00359-005-0048-7 16341863

[B136] Zou H. S. Juan C. C. Chen S. C. Wang H. Y. Lee C. Y. (2003). Dopaminergic regulation of crustacean hyperglycemic hormone and glucose levels in the hemolymph of the crayfish *Procambarus clarkii*. *J. Exp. Zool. Part A Comp. Exp. Biol.* 298 44–52. 10.1002/jez.a.10273 12840838

