# Supplementary figures and images for: Immunocytochemical Localization of Enzymes Involved in Dopamine, Serotonin, and Acetylcholine Synthesis in the Optic Neuropils and Neuroendocrine System of Eyestalks of Paralithodes camtschaticus
Source: Front Neuroanat. 2022 Apr 8;16:844654. doi: 10.3389/fnana.2022.844654 (PMC9024244; doi:10.3389/fnana.2022.844654)

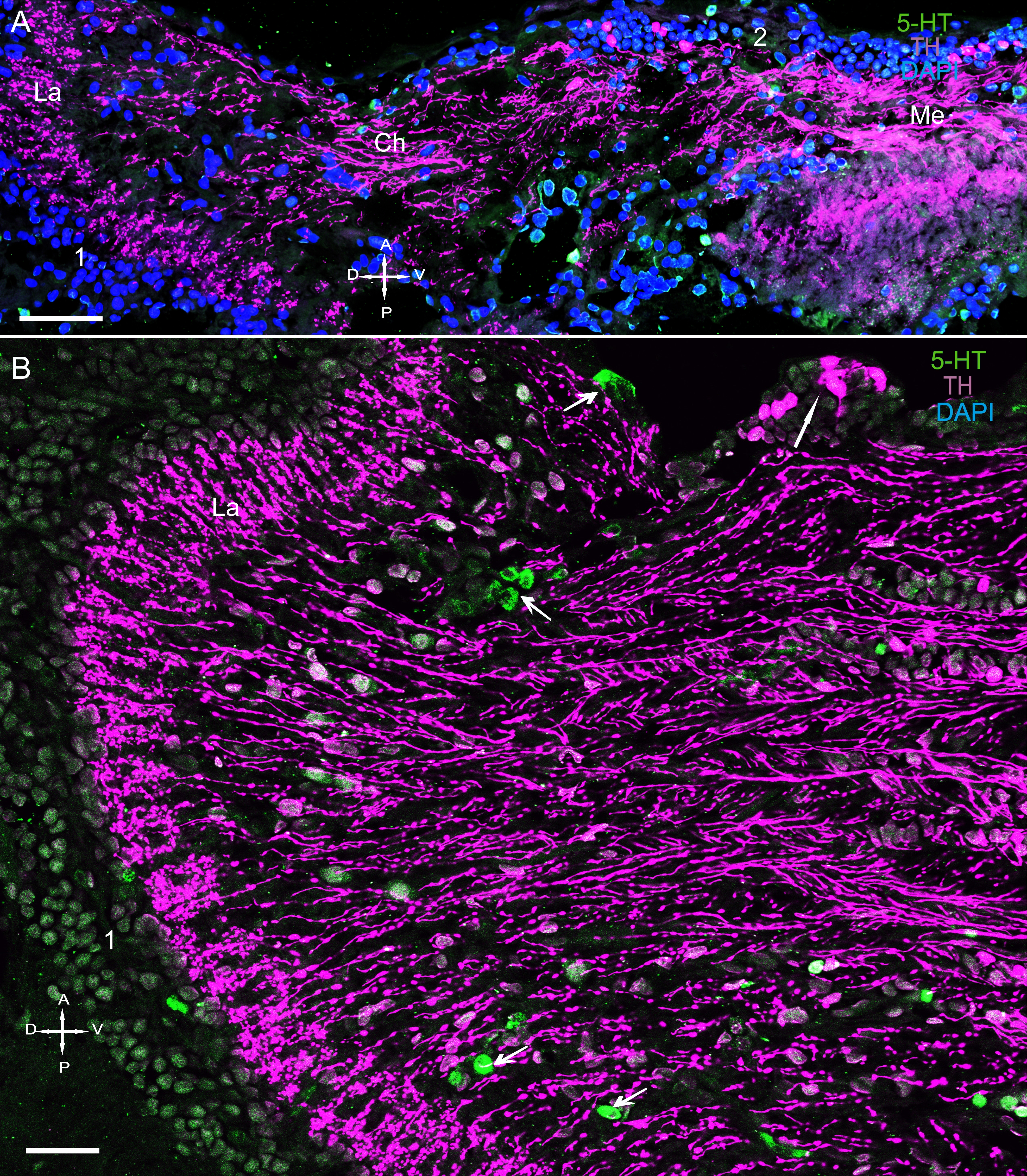

Supplement: Supplementary Figure 1 — Immunolocalization of serotonin (5-HT) and tyrosine hydroxylase (TH) in the optic neuropil lamina of Paralithodes camtschaticus. (A) TH-positive nerve fibers between the lamina and the medulla form the first optic chiasma. (B) Varicose processes of TH-immunoreactivity in the lamina of a plexiform layer and at the first chiasma. Arrows indicate 5-HT in the amacrine neurons; large arrows indicate TH-positive neurons localized at the first chiasma. Abbreviations: La, lamina; 1, cell clusters; D, dorsal; V, ventral; L, lateral; M, medial; A, anterior; P, posterior. Scale bars = 100 μm. [file Image_1.JPEG]

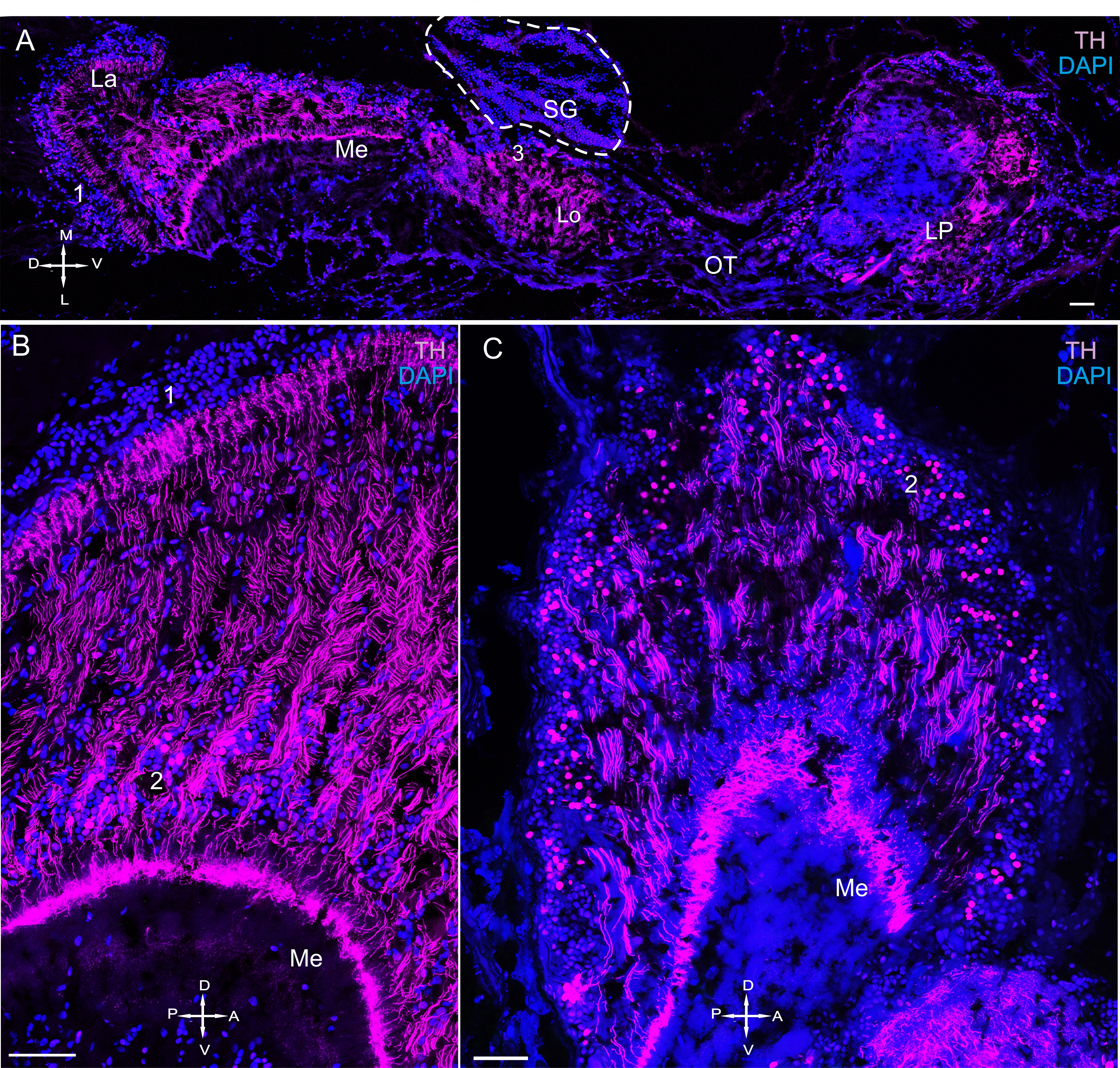

Supplement: Supplementary Figure 2 — Immunolocalization of tyrosine hydroxylase (TH) in the optic neuropils of Paralithodes camtschaticus. (A) Horizontal section showing the immunohistochemical localization of TH in the optic neuropils (HN) and lateral protocerebrum; (B,C) Immunohistochemical localization of TH in the neurons and nerve fibers of the medulla. Abbreviations: La, lamina; Me, medulla; Lo, lobula; SG, sinus gland; OT, optic tract; LP, the lateral protocerebrum; D, dorsal; V, ventral; L, lateral; M, medial; A, anterior; P, posterior. Scale bar = 100 μm. [file Image_2.JPEG]
